# Impact of Early Subcutaneous Basal Insulin With Intravenous Insulin Infusion for Diabetic Ketoacidosis and Glycaemic Outcomes: A Systematic Review and Meta‐Analysis

**DOI:** 10.1002/edm2.70277

**Published:** 2026-07-06

**Authors:** Abdallah AbuJlambo, Maha AbuZarifa, Rejean R. R. Sawh, Subhash Reddy Narayana Reddy N, Muhammad Bin Salman, Insiya Mohammed Rampurawala, Yara Ashour, Hazem Ayesh

**Affiliations:** ^1^ Department of ICU Al‐Shifa Hospital Gaza Palestine; ^2^ Al‐Azhar Branch Al‐Quds University Gaza Palestine; ^3^ UCHealth Parkview Medical Center Pueblo Colorado USA; ^4^ Department of Public Health Bruhat Bengaluru Mahanagara Palike (BBMP) Karnataka India; ^5^ Department of Internal Medicine Rawalpindi Medical University Rawalpindi Pakistan; ^6^ JSS Medical College Mysore India; ^7^ Al‐Quds University Gaza Palestine; ^8^ Deaconess Health System Evansville Indiana USA

**Keywords:** basal insulin, diabetic ketoacidosis, glycaemic control, intravenous insulin, meta‐analysis, randomised controlled trials

## Abstract

**Background:**

Diabetic ketoacidosis (DKA) is a life‐threatening metabolic emergency requiring prompt insulin therapy. Although intravenous (IV) insulin infusion remains the standard treatment, early initiation of subcutaneous (SC) basal insulin during IV therapy may improve outcomes. We evaluated the efficacy and safety of early basal insulin administration in adults with DKA.

**Methods:**

A systematic review and meta‐analysis following PRISMA 2020 guidelines searched PubMed, Embase and Cochrane Library from inception to February 2026 for randomised controlled trials (RCTs) comparing early SC basal insulin plus IV insulin versus IV insulin alone in adults with DKA. Primary outcomes were time to DKA resolution and hospital length of stay. Secondary outcomes included total fluid requirement, hypoglycaemia, rebound hyperglycaemia, recurrent DKA, electrolyte disturbances and mortality. Meta‐regression explored sources of heterogeneity.

**Results:**

Eight RCTs involving 407 participants were included. Early basal insulin significantly reduced time to DKA resolution (MD −4.06 h, 95% CI −5.53 to −2.58; *p* < 0.0001; *I* ^2^ = 56.3%), with consistent findings in sensitivity analysis (MD −3.44 h; *I* ^2^ = 0%). Total fluid requirements decreased by approximately 400 mL, although clinical significance was modest. No significant differences were observed in hospital length of stay, rebound hyperglycaemia, recurrent DKA, hypoglycaemia, hypokalaemia, or mortality. Meta‐regression showed no significant effect modification by age, BMI, sample size, or male proportion.

**Conclusions:**

Early SC basal insulin combined with IV insulin significantly shortens DKA resolution time and modestly reduces fluid requirements without increasing adverse events. While no significant effects were observed on hospital stay or recurrence outcomes, these findings support the safety and potential clinical benefit of early basal insulin use in DKA management. Larger multicentre trials are warranted.

## Graphical abstract

Early subcutaneous basal insulin initiation alongside intravenous insulin significantly shortened diabetic ketoacidosis resolution time and modestly reduced fluid requirements without increasing adverse events, supporting its potential role as a safe adjunctive strategy in adult DKA management.

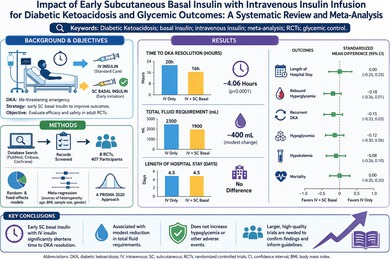

## 1 Introduction

Diabetic ketoacidosis (DKA) is the most life‐threatening acute complication of diabetes mellitus with a high mortality risk. It presents as hyperglycaemia, ketonaemia and metabolic acidosis. DKA continues to be a leading cause of hospital admissions and morbidity among adult patients with diabetes, particularly adults with type 1 diabetes and insulin‐dependent type 2 diabetes [1]. A recent study in the United States showed that from 2014 to 2017, the aggregated charges among DKA patients increased from $5.28 billion to $6.76 billion [2]. DKA drives higher readmission rates, disproportionately affecting lower‐income populations. Preventing it improves quality of life and reduces the strain on the healthcare system [3]. The standard of care for DKA includes the administration of intravenous (IV) fluids, a continuous infusion of IV regular insulin and careful electrolyte replacement. Once the ketoacidosis resolves, the patient should be transitioned to subcutaneous insulin [4].

The current American Diabetes Association guidelines recommend an overlap of at least 2–4 h while transitioning from IV to SC insulin therapy to avoid gaps in insulin activity [5]. However, endocrinology fellows rotating on the hospital Diabetes Consult Service (DCS) had observed practice patterns during consults for post‐DKA management where there was less than 2 h or sometimes no overlap between IV and SC insulin [6]. Thus, increasing the risk for hyperglycaemia, ketone re‐accumulation and an extended hospital stay [7]. Recent interest has focused on the potential benefits of early initiation of long‐acting insulin analogues, such as insulin glargine, insulin detemir, or insulin degludec, in the early phase of DKA, while the patient is on IV insulin. Recent studies showed that starting long‐acting (basal) insulin early in the course of treatment leads to shorter treatment periods of hospital stay [8, 9].

Early initiation also decreases the length of time until DKA resolution occurs and provides a smoother transition to using outpatient insulin with minimal risk of hypoglycaemia. A retrospective analysis showed that early basal insulin was associated with 13.8 h shorter continuous intravenous infusion time with a trend toward lower rates of hypoglycaemia [10].

Many of the available studies are limited by small sample sizes and inconsistent findings. Given the clinical importance of DKA and its association with increased mortality, prolonged hospital stay and significant resource utilisation, we planned to conduct a systematic review and meta‐analysis. This approach would allow pooling of data across studies to provide more robust estimates and may help inform guideline development and support clinical decision‐making regarding the use of early subcutaneous insulin in the management of DKA.

## 2 Methods

### 2.1 Study Design

This systematic review and meta‐analysis followed the PRISMA 2020 statement [11] and has been prospectively registered with PROSPERO as a previously defined methodology. Registration number CRD420251158990 [8].

### 2.2 Eligibility Criteria

PICOS criteria applied to each study are as follows: Randomised controlled trials (RCTs) study design that focuses on adults ≥ 18 years or older with DKA. Eligible studies were randomised controlled trials comparing early administration of basal insulin (long‐acting or ultra–long‐acting insulin analogues, including insulin glargine and insulin degludec) initiated during the active phase of diabetic ketoacidosis (DKA) defined as administration during ongoing intravenous insulin infusion or prior to biochemical resolution versus standard care consisting of intravenous insulin infusion alone or intravenous insulin with basal insulin administered only as bridging therapy after DKA resolution (typically 1–2 h before discontinuation of intravenous insulin). However, the comparator was standard DKA management (relative to standard DKA treatment, which is IV insulin alone without the early initiation of basal insulin or placebo). Primary outcomes were Time to resolution of DKA and length of hospital stay. Secondary outcomes: hypoglycaemia, rebound hyperglycaemia, recurrent DKA, total intravenous fluid requirement, electrolyte disturbances (e.g., hypokalaemia) and all‐cause mortality. Total intravenous fluid volume was extracted as reported in each study and reflected the cumulative fluids administered during the acute DKA management period. Definitions varied across studies, most commonly from admission to DKA resolution or hospital discharge. All observational studies, non‐randomised trials, uncontrolled trials, conference abstracts without full article data, editorials/letters, post hoc analysis with new outcome data and included only children were excluded from this review.

### 2.3 Search Strategy

We have searched PubMed, Embase and the Cochrane Library from the beginning of the database until 18th February 2026. The search utilised Medical Subject Headings (MeSH) and free‐text keywords connected using Boolean operators, either AND or OR. Key search terms included: “diabetic ketoacidosis”, “DKA”, “basal insulin”, “long‐acting insulin”, “insulin glargine”, “insulin degludec”, “early insulin”, “subcutaneous insulin”, “randomized controlled trial”, and “adult”. There were no restrictions on the language or publication status of the records searched. Additional relevant studies not included in the database were identified manually by reviewing the reference lists of all studies deemed relevant from our original search, including those listed in the related systematic reviews.

### 2.4 Study Selection

Each investigator independently screened the title and abstract of each identified record to ensure eligibility. Two independent reviewers (A.A. and M.A.) conducted study selection and data extraction. Discrepancies were resolved through discussion and consensus and a third reviewer (H.A.) was consulted when needed. Each full‐text article from each potentially relevant study was then read and assessed by the two investigators for final inclusion. Disagreements were resolved by consensus.

### 2.5 Data Extraction

Data extraction was carried out utilising a pre‐tested standard data collection form. The data extracted included study design (author, year and site), methods, sample size, demographics of participants, initial severity of diabetic ketoacidosis, description of intervention and comparison groups (type of basal insulin and amount, plus when basal insulin was started), follow‐up and outcomes. Outcome data, such as counts and summary statistics required for the meta‐analysis, were gathered across all relevant outcomes. If incomplete or inconsistent data were found, data were estimated from the available data. No access to individual patient‐level data was available. When necessary, missing data were estimated using standard statistical methods recommended by the Cochrane Handbook, such as deriving means and standard deviations from medians, ranges, or interquartile ranges. These estimations were based solely on published summary data, and no re‐analysis of original datasets was performed. Sensitivity analyses were conducted to assess the impact of these estimations on pooled outcomes, and no material differences were observed.

### 2.6 Quality Assessment

Reviewers independently assessed risks of bias using the Cochrane Risk of Bias Tool for Randomised Trials (RoB 2) [12]. Five domains were evaluated as follows: (1) Bias arising from the randomisation process, (2) Deviations from intended interventions, (3) Missing outcome data, (4) Measurement of the outcome, (5) Selection of the reported result. Each category was assigned a risk level of low, high, or some concerns. We determined the final overall rating in accordance with Cochrane guidance, settling any differences of opinion through mutual agreement [13]. Additionally, the Grading of Recommendations, Assessment, Development and Evaluation (GRADE) system [14] was used to assess the overall certainty of evidence using the GRADEpro tool [15]. The certainty of evidence was rated across four levels: high, moderate, low and very low.

### 2.7 Statistical Analysis

Meta‐analytic procedures utilised R software (version 4.5.0; GUI 1.81, Big Sur ARM build) [16]. Meta‐analysis is considered quantitative when at least 2 studies report the same outcome. Continuous outcomes such as time to DKA resolution, length of hospital stay, volume of fluid and total insulin dose were analysed and presented as mean differences (MDs) with 95% confidence intervals (CIs). Dichotomous (binary) outcomes, including hypoglycaemia, rebound hyperglycaemia, recurrent DKA and in‐hospital mortality, were analysed using risk ratios (RRs) with 95% confidence intervals (CIs). To pool the data, a fixed‐effect meta‐analytic model was used when statistical heterogeneity was low or insignificant. However, if there was evidence of substantial heterogeneity, we applied a random‐effects model instead. Cochran's Q Test for statistical heterogeneity and *I* ^2^ to represent the level of statistical heterogeneity, statistical heterogeneity was defined as low (25% or less), moderate (between 25% and 50%) and high (greater than 75%). Sensitivity analyses were performed to evaluate the robustness of the pooled results. These included leave‐one‐out analyses and comparisons between fixed‐effect and random‐effects models. In addition, meta‐regression analyses were conducted to explore potential sources of heterogeneity. Publication bias was not formally assessed using funnel plots or statistical tests (e.g., Egger's test), as fewer than 10 studies were included in the meta‐analysis for each outcome. According to Cochrane recommendations, such methods are not reliable when the number of studies is small due to low statistical power. In addition, in case there is high heterogeneity for the outcome, we conducted meta‐regression to explore the potential source of heterogeneity. Moderator factors included male percentage, BMI, Age and sample size. QM refers to the test statistic used in meta‐regression to assess the significance of moderator variables.

## 3 Result Section

**FIGURE 1:**
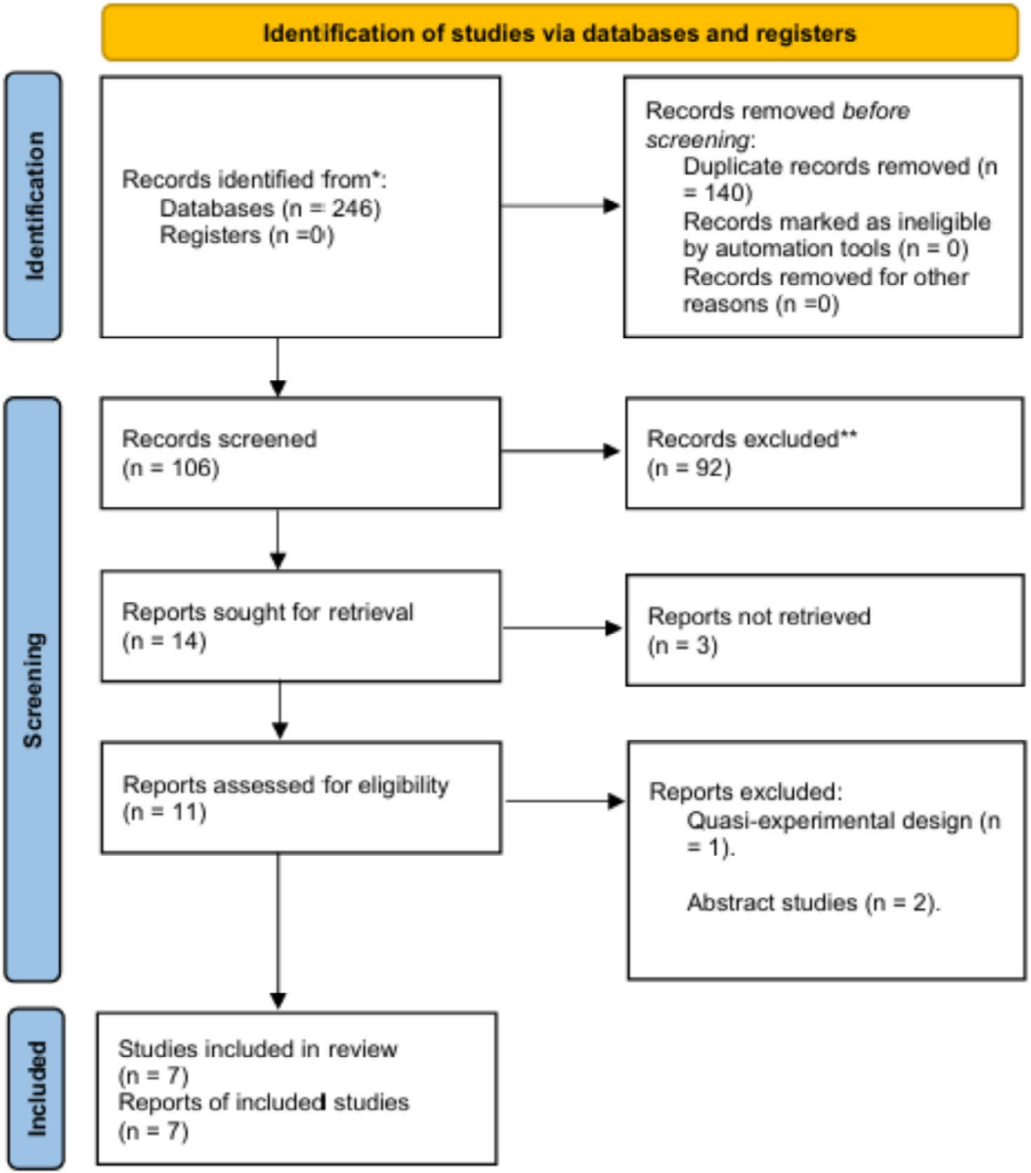
PRISMA 2020 flow diagram for study identification and selection.

A systematic search identified studies, of which 351 were from databases including PubMed, Embase and coherence. There were 106 removed from screening because they were duplicates. The final included studies were eight studies that satisfied the predefined inclusion parameters, and all of them were incorporated into the meta‐analysis. The selection process is detailed in Figure 1, presenting the PRISMA flow diagram showing the study selection process. Eight studies were ultimately included in the systematic review and meta‐analysis.

### 3.1 Study Characteristics

Our systematic review and meta‐analysis included eight studies [8, 17, 18, 19, 20, 21, 22, 23] from 2011 to 2026 that enrolled DKA patients, with a sample size ranging from 25 to 90. Detailed baseline clinical and biochemical characteristics of the intervention and control groups are presented in Table 1. Overall, reporting of baseline comparability between groups was limited across the included studies. For age, the mean age ranged from 29 to 58 years; most studies included patients with type 1 and type 2 diabetes. Laboratory investigations showed severe hyperglycaemia (blood glucose 497–605 mg/dL), acidosis (pH 7.09–7.31, HCO3 6.34–12.8 mEq/L), where β‐hydroxybutyrate was only reported in two studies. The most commonly reported precipitating factors for DKA included infection and insulin non‐adherence. The key study characteristics, including study design, sample size and patient demographics, are summarised in Table 2.

**TABLE 1:** Baseline clinical and biochemical characteristics of patients included in the intervention and control groups.

Study ID	Study design	Country	Population DM type	Inclusion criteria	Exclusion criteria	Treatment (intervention vs. control)	Mean age	SD age	Total *N*
Assaad Khalil (2012)	RCT	Egypt	N/A	Age 15–60, blood sugar > 250 mg/dL, pH < 7.25, Bicarbonate < 15 mmol/L, AG > 12 mmol/L	Hemodynamic instability, Congestive heart failure and Renal impairment	Glargine 0.3 U/kg SC 6 h from treatment + Standard DKA treatment vs. Standard DKA treatment	N/A	N/A	40
Hsia (2012)	RCT	USA	Type1, Type 2	Known diagnosis of diabetes receiving IV insulin infusion and lucent mentally to sign a consent form	Newly diagnosed hyperglycaemia or critical illness and pregnant women	Glargine 0.25 U/kg SC 12 h from RI infusion + Standard DKA treatment vs. Standard DKA treatment	N/A	N/A	N/A
Doshi (2015)	RCT	USA	Type1, Type 2	Blood sugar > 200 mg/dL, pH < 7.3, HCO_3_ < 18 mg/dL, ketonaemia or ketonuria and AG > 16	Patients who required IV inotropic resuscitation including use of vasopressors, were pregnant, had end‐stage renal disease, were under 18 years of age, were unwilling to consent to participate in the trial, were currently prisoners, were transferred to another hospital, or required emergent surgery	Glargine 0.3 U/kg SC 2 h from diagnosis + Standard DKA treatment vs. Standard DKA treatment	39.42	14.32	40
Houshyar (2015)	RCT	Iran	Type1, Type 2	Definitive diagnosis of DKA according to the American Diabetes Association criteria, age > 12 years and consent to participate	Persistent hypotension (SBP < 80 mmHg in spite of receiving 1000 cc saline normal), acute MI, progressive renal or hepatic failure, the need for emergency surgery, pregnancy and the lack of consent for participation	Glargine 0.4 U/kg SC 3 h from RI infusion + Standard DKA treatment vs. Standard DKA treatment	29.45	14.49	40
Ammar (2023)	RCT	Egypt	Type1, Type 2	DKA diagnosis, age 18–70 years old, Type I or Type II diabetes mellitus, both men and women. Receiving oral or injectable hypoglycaemic medication. Diabetes has been present for over 5 years in both surgical and medical cases	Severe persistent hypotension, end‐stage renal disease or progressive renal failure, acute myocardial infarction, pregnancy and liver cell failure	Glargine 0.19–0.27 U/kg SC 2 h from ICU admission + Standard DKA treatment vs. Standard DKA treatment	31.56	7.33	52
Thammakosol (2023)	Single‐centre, open‐label RCT	Thailand	Type 1 (Classic & LADA), Type 2, & Others (pancreatic diabetes, nivolumab‐induced diabetes, post‐transplant diabetes)	Adults ≥ 18 years with DKA by ADA criteria	History of insulin glargine allergy, haemodynamic instability requiring vasopressor, pregnancy or breastfeeding	Glargine 0.30 U/kg SC 3 h from diagnosis + Standard DKA treatment vs. Standard DKA treatment	56.2	16.5	60
El Feky (2025)	RCT	Egypt	Type 1	ADA recommendations of DKA diagnosis	Pregnant and breastfeeding females, type 2 diabetes mellitus, advanced renal or hepatic disease and hemodynamic instability	Glargine U‐100 or glargine U‐300 0.3 U/kg SC 3 h from diagnosis + Standard DKA treatment vs. Standard DKA treatment	27.21	7.99	90
Thammakosol (2025)	RCT	Thailand	Type 1, Type 2, LADA, Immune check point inhibitor induced DM, pancreatic DM, new onset diabetes after transplantation	Age 18 years or older, DKA based on ADA statements	Plasma glucose levels lower than 250 mg/dL, history of insulin allergy, haemodynamic instability, pregnancy, breastfeeding and those planning to receive a SC treatment regimen for mild DKA	Degludec 0.30 U/kg SC 3 h from diagnosis + Standard DKA treatment vs. Standard DKA treatment	55.9	17.81	80

**TABLE 2:** Characteristics of studies included in the systematic review and meta‐analysis.

Study (year)	Sample size (IG/CG)	Age: mean ± SD (IG vs. CG)	Male % (IG vs. CG)	BMI, kg/m^2^: mean ± SD (IG vs. CG)	HbA1c, %: mean ± SD (IG vs. CG)	pH: mean ± SD (IG vs. CG)	Bicarbonate, mEq/L: mean ± SD (IG vs. CG)	Blood Glucose, mg/dL: mean ± SD (IG vs. CG)	β‐Hydroxybutyrate: mean ± SD (IG vs. CG)	Potassium, mEq/L: mean ± SD (IG vs. CG)	Serum creatinine, mg/dL: mean ± SD (IG vs. CG)	Precipitating factors
Assaad Khalil (2012)	30/15	N/A	N/A	N/A	N/A	7.12 ± 0.10 vs. 7.13 ± 0.11	6.5 ± 3.6 vs. 7.2 ± 3.4	598.0 ± 103.8 vs. 626.2 ± 121.7	N/A	N/A	N/A	N/A
Hsia (2012)	12/13	N/A	41.7 vs. 46.2	N/A	11.0 ± 2.2 vs. 12.1 ± 1.9	N/A	N/A	N/A	N/A	N/A	N/A	N/A
Doshi (2015)	20/20	38.5 ± 11.17 vs. 40.33 ± 17.15	70 vs. 50	N/A	N/A	7.20 ± 0.16 vs. 7.17 ± 0.16	11.67 ± 5.19 vs. 11.83 ± 4.39	605.5 ± 178.72 vs. 548 ± 199.46	N/A	N/A	1.70 ± 0.87 vs. 1.13 ± 0.40	Infection, new diagnosis with initial presentation, noncompliance
Houshyar (2015)	20/20	29.65 ± 13.60 vs. 29.25 ± 15.69	45 vs. 45	21.06 ± 2.92 vs. 22.29 ± 3.42	12.31 ± 2.40 vs. 12.78 ± 2.41	7.09 ± 0.15 vs. 7.09 ± 0.14	6.34 ± 1.76 vs. 6.34 ± 1.76	540 ± 208.8 vs. 497.34 ± 102.6	N/A	4.65 ± 0.74 vs. 4.59 ± 0.59	0.13 ± 0.03 vs. 0.13 ± 0.03	Newly identified cases, non‐adherence to DM control modalities and inappropriate use of insulin, acute pancreatitis and pyelonephritis, pneumonia, infection of diabetic foot, common cold, sinusitis, pyelonephritis and gastroenteritis
Ammar (2023)	26/26	33.12 ± 7.83 vs. 30.0 ± 6.57	46.2 vs. 53.8	N/A	N/A	N/A	N/A	N/A	N/A	N/A	N/A	Medical, Surgical
Thammakosol (2023)	30/30	54.2 ± 14.3 vs. 58.2 ± 18.5 years	43.3 vs. 36.7	21.8 ± 4.2 vs. 22.4 ± 3.6	12.77 ± 2.72 vs. 11.54 ± 3.64	7.20 ± 0.15 vs. 7.31 ± 0.16	10.3 ± 5.1 vs. 12.8 ± 5.2	601.5 ± 210.2 vs. 554.6 ± 193.5	9.3 ± 3.2 vs. 6.5 ± 3.0	5.05 ± 1.14 vs. 4.50 ± 0.92	1.17 ± 0.61 vs. 1.12 ± 0.71	Infection, Discontinue medication, Inadequate medication, Stroke, First diagnosis diabetes, Others (alcohol abuse, glucocorticoid use, thyroid storm, surgery)
El‐Feky (2025)	60/30	28.18 ± 8.66 vs. 28.18 ± 8.67	56.7 vs. 53.3	24.34 ± 2.96 vs. 25.42 ± 3.06	9.21 ± 1.54 vs. 9.23 ± 1.76	7.16 ± 0.13 vs. 7.17 ± 0.14	7.38 ± 3.29 vs. 7.95 ± 4.03	528.1 ± 96.52 vs. 558.83 ± 72.36	N/A	4.83 ± 0.84 vs. 4.91 ± 0.81	1.08 ± 0.36 vs. 1.12 ± 0.35	Missed doses Infection First diagnosis DM
Thammakosol (2025)	40/40	55.4 ± 15.8 vs. 56.4 ± 19.8	42.5 vs. 42.5	23.4 ± 5.0 vs. 22.9 ± 6.0	11.6 ± 2.4 vs. 10.9 ± 3	7.24 ± 0.15 vs. 7.28 ± 0.1	10.6 ± 5.0 vs. 11.1 ± 4.9	504.4 ± 148.3 vs. 512.9 ± 210.0	7.3 ± 3.0 vs. 6.7 ± 2.9	4.77 ± 0.74 vs. 4.61 ± 1.03	0.89 ± 0.29 vs. 1.11 ± 0.79	Infection, Discontinue medication, Inadequate medication, Myocardial infarction, Stroke, Pancreatitis, First DM diagnosis

### 3.2 Quality Assessment

**FIGURE 2:**
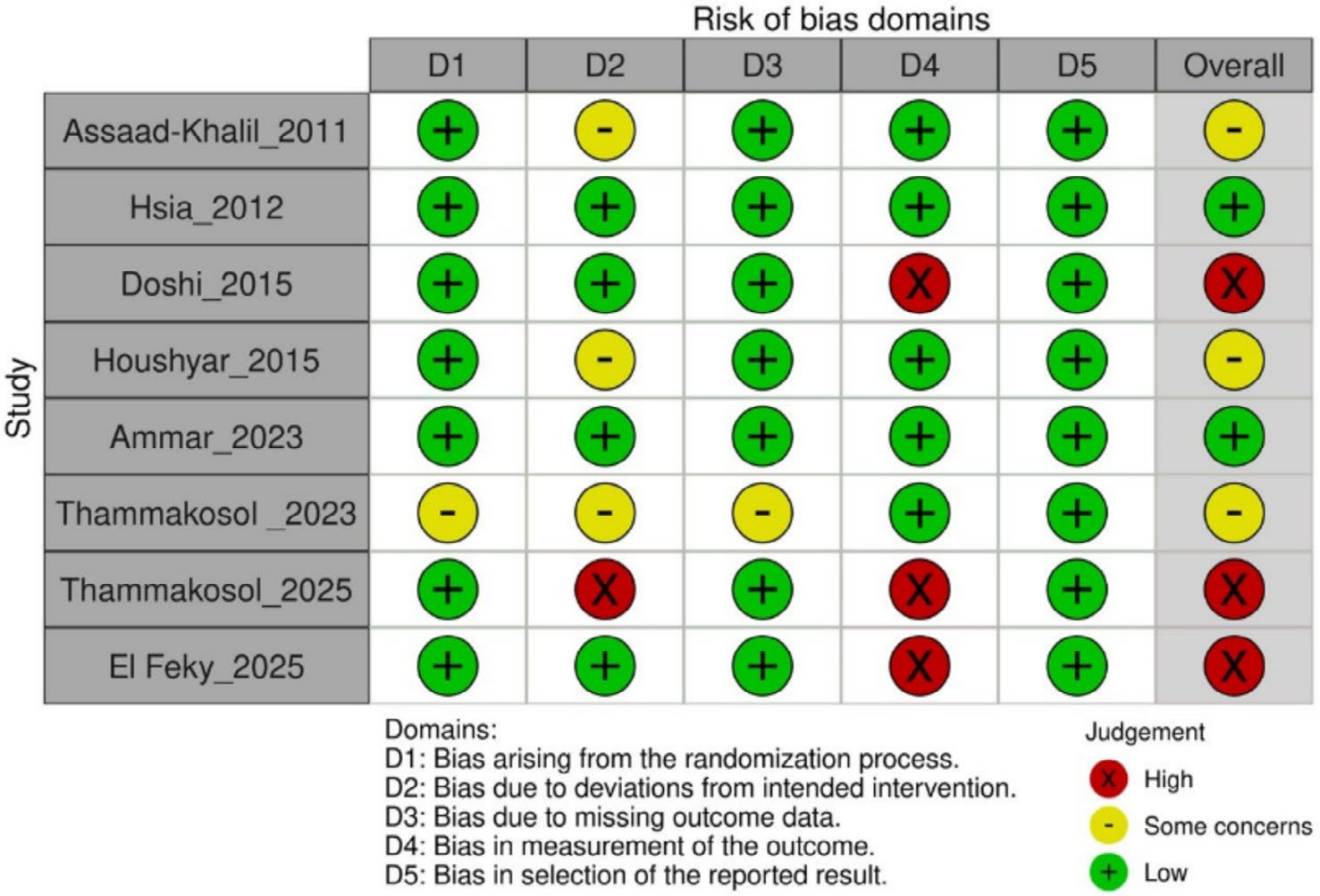
Risk of bias assessment of randomised controlled trials using the Cochrane Risk of Bias 2 (RoB 2) tool.

Risk of bias was assessed using the Cochrane Risk of Bias 2 (RoB 2) tool across the five domains (Figure 2). Overall, two studies were judged to have a low risk of bias across all domains, while three studies were assessed as having a high overall risk of bias, primarily driven by deviations from intended interventions (D2) and concerns in outcome measurement (D4). The remaining studies demonstrated some concerns, most commonly related to incomplete reporting of randomisation procedures and selective outcome reporting. Bias related to bias arising from the randomisation process (D1), missing outcome data (D3) and selective reporting (D5) was generally low across most included studies.

### 3.3 Effect of Early Subcutaneous Basal Insulin Use for DKA Management

#### 3.3.1 Time to DKA Resolution

**FIGURE 3:**
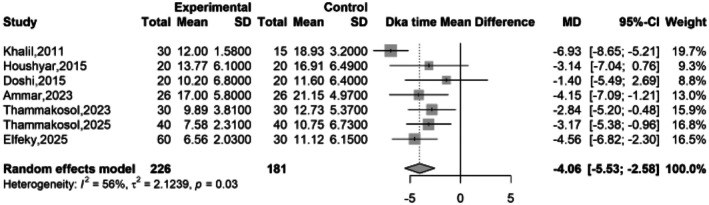
Effect of early basal insulin versus standard care on time to resolution of diabetic ketoacidosis (DKA): Forest plot of mean difference (MD) with 95% confidence intervals (CI).

**FIGURE 4:**
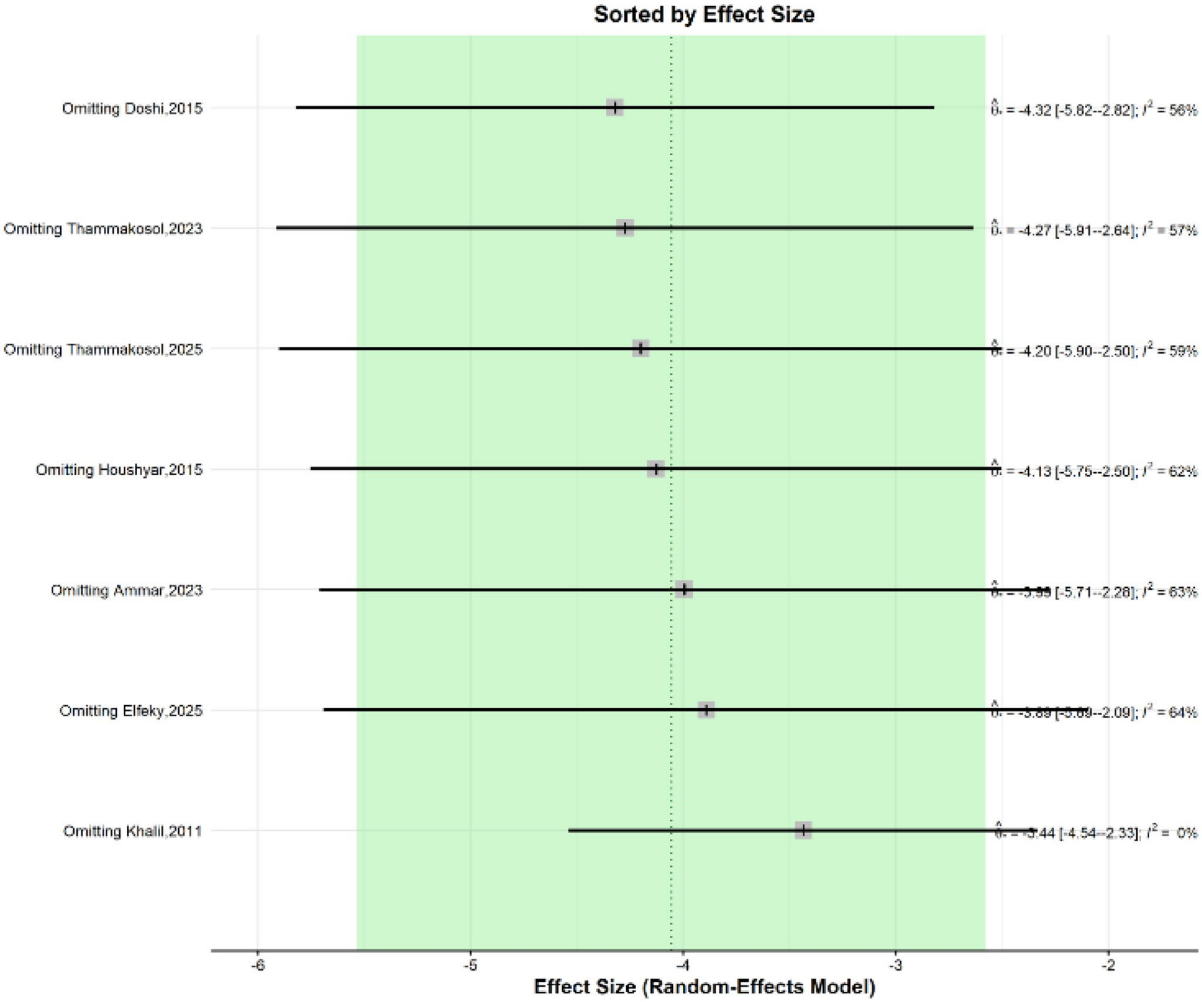
Sensitivity analysis (leave‐one‐out) for time to resolution of DKA after exclusion of the Assaad‐Khalil 2011 study: Forest plot of mean difference (MD) with 95% confidence intervals (CI).

Seven studies, including 407 participants, evaluated the intended outcome. Given the absence of statistical heterogeneity, a random‐effect meta‐analysis was employed to pool the risk ratios. The pooled estimated mean differences showed the time needed to resolve DKA was much shorter in the basal insulin than the standard care, with about 4.06 h (95% CI: −5.53 to −2.58, *p* < 0.0001). There was moderate heterogeneity (*I* ^2^ = 56.3%, *p* = 0.03), suggesting some variability in the effect size among studies (Figure 3). The observed heterogeneity may be attributed to differences in the timing of basal insulin initiation, baseline severity of DKA and study‐specific protocols. Sensitivity analysis after removing the Assaad‐Khallil study revealed that early basal insulin significantly decreased the time for DKA resolution, with 3.44 h in comparison to standard DKA treatment (*I* ^2^ = 0%; Figure 4). Meta‐regression analysis showed no significant moderator effects for sample size, proportion of male participants, age and BMI. None of the covariates (sample size, male proportion, age, or BMI) was significantly associated with the intended outcome (all *p* > 0.05; Figure S5). The adjusted estimates remained similar to the unadjusted estimate (QM = 1.99, *p* = 0.37). The certainty of evidence was generally moderate due to concerns of risk of bias (Table S1).

#### 3.3.2 Total Amount of Fluid

**FIGURE 5:**
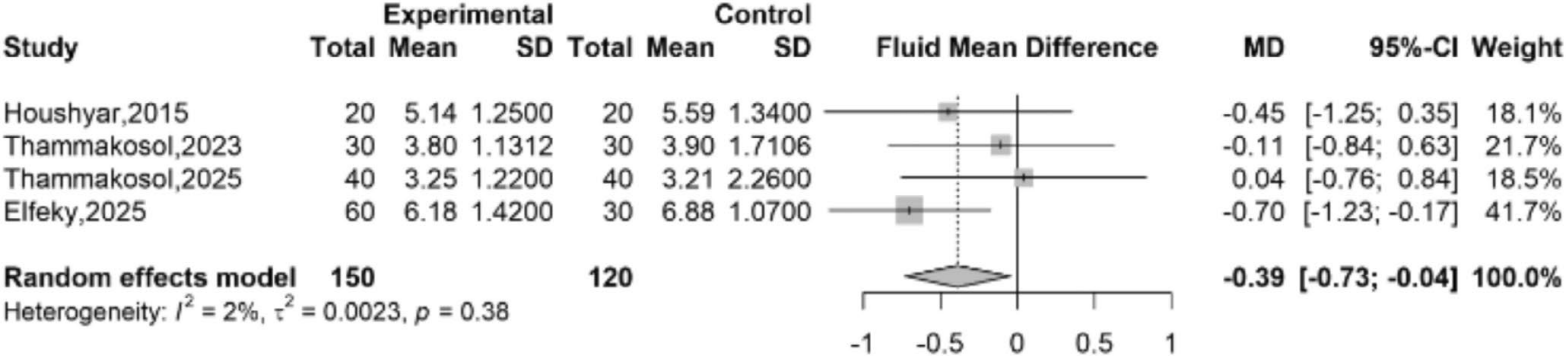
Forest plot comparing early basal insulin versus standard care for total intravenous fluid requirement in DKA (mean difference).

Four studies reported a total amount of fluid (Figure 5). A random‐effect meta‐analysis showed that early basal insulin decreases the amount of fluid by around 400 mL per patient. The heterogeneity was neglected for this outcome (*I* ^2^ 1.8%). The meta‐regression for the total amount of fluid did not find any significant moderator effects. None of the covariates (male proportion, age, BMI, or sample size) was significantly associated with the total amount of fluid (all *p* > 0.05). The adjusted estimates remained similar to the unadjusted estimate (QM = 0.96, *p* = 0.62; Figure S6).

#### 3.3.3 Length of Hospital Stay

**FIGURE 6:**
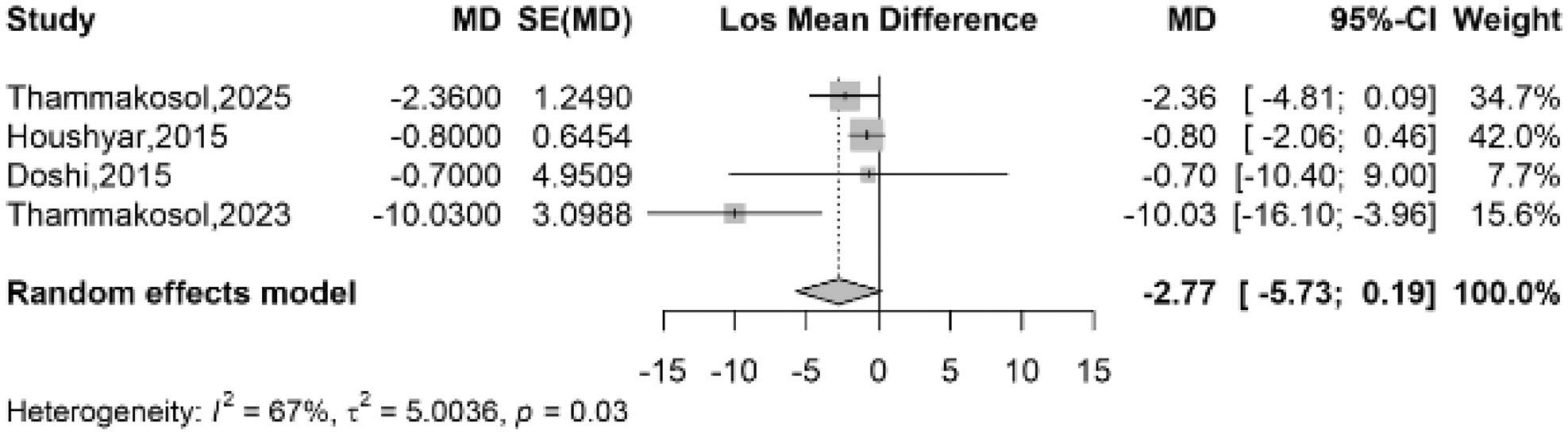
Forest plot comparing early basal insulin versus standard care for length of hospital stay in DKA (mean difference, days).

**FIGURE 7:**
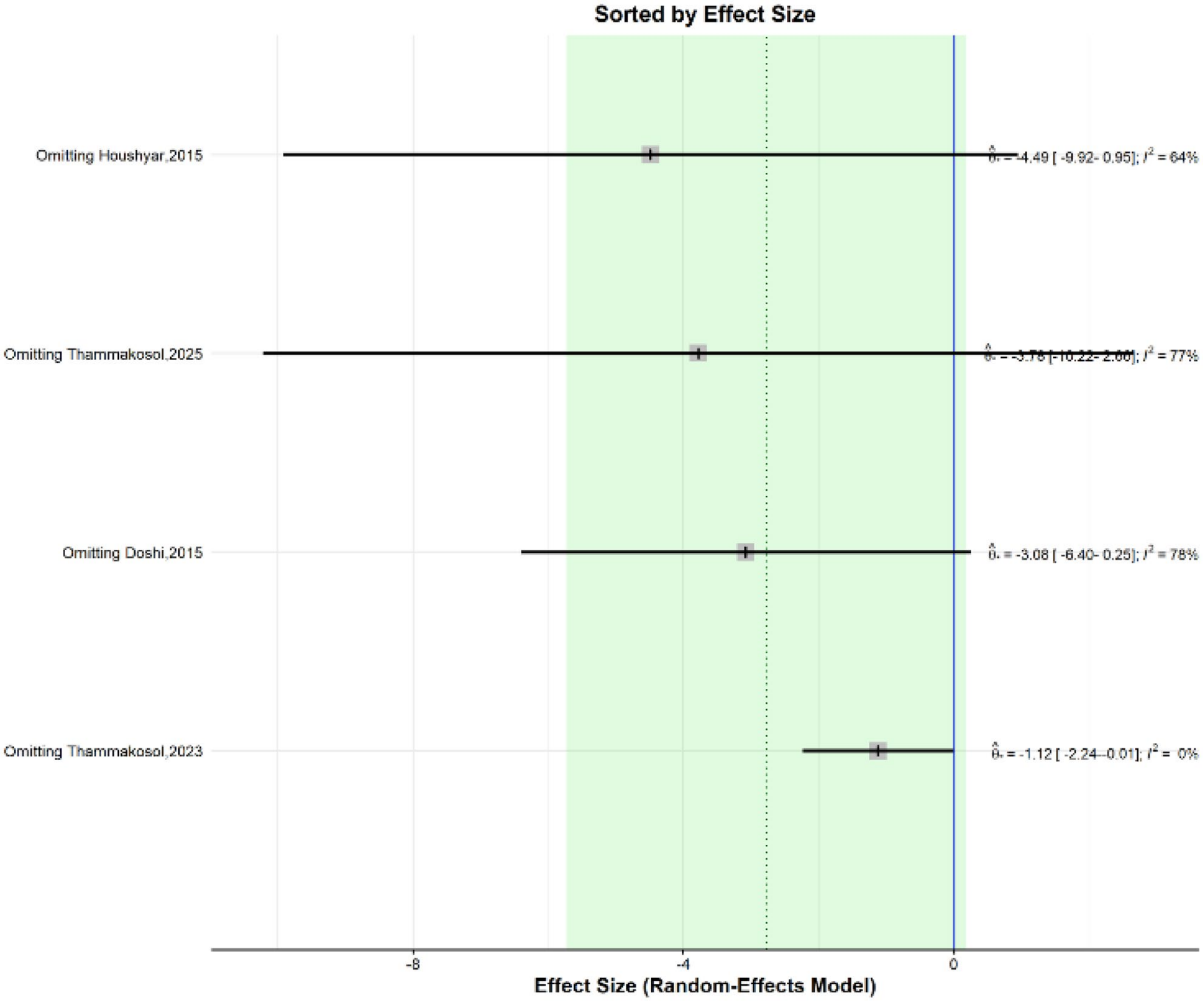
Forest plot of sensitivity analysis (leave‐one‐out) for length of hospital stay after exclusion of the Thammakosol 2023 study.

Four studies included 217 participants and reported data on the length of hospital stay. A random effects model was conducted to estimate the mean difference, which revealed that early basal insulin decreases the length of hospital stay with a RR of −2.77 (95% CI: −5.73 to 0.19, *p* = 0.066). Although the reduction in length of stay did not reach statistical significance, the point estimate suggests a potentially clinically meaningful reduction and a substantial benefit cannot be excluded due to imprecision. Figure 6 illustrates the forest plot for this outcome. Based on substantial heterogeneity (*I* ^2^ = 67.5%, *p* = 0.0264), sensitivity analysis was conducted, where we removed the Thammakosol 2023; the heterogeneity decreased to *I* ^2^ = 0.0%, indicating minimal variability between studies after removing the outlier (Figure 7). The pooled MD was −1.04 days (95% CI: −2.84 to 0.75) with 160 participants, which remained non‐significant (*p* = 0.51). The meta‐regression for length of hospital stay did not find any significant moderator effects. None of the covariates (male proportion, age, BMI, or sample size) was significantly associated with length of hospital stay (all *p* > 0.05; Figure S7) The certainty of evidence was generally low due to concerns of risk of bias and imprecision (Table S1).

#### 3.3.4 Rebound Hyperglycaemia

**FIGURE 8:**
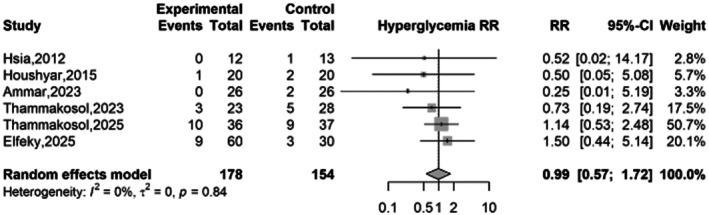
Forest plot comparing early basal insulin versus standard care for risk of rebound hyperglycaemia in DKA (risk ratio).

**FIGURE 9:**
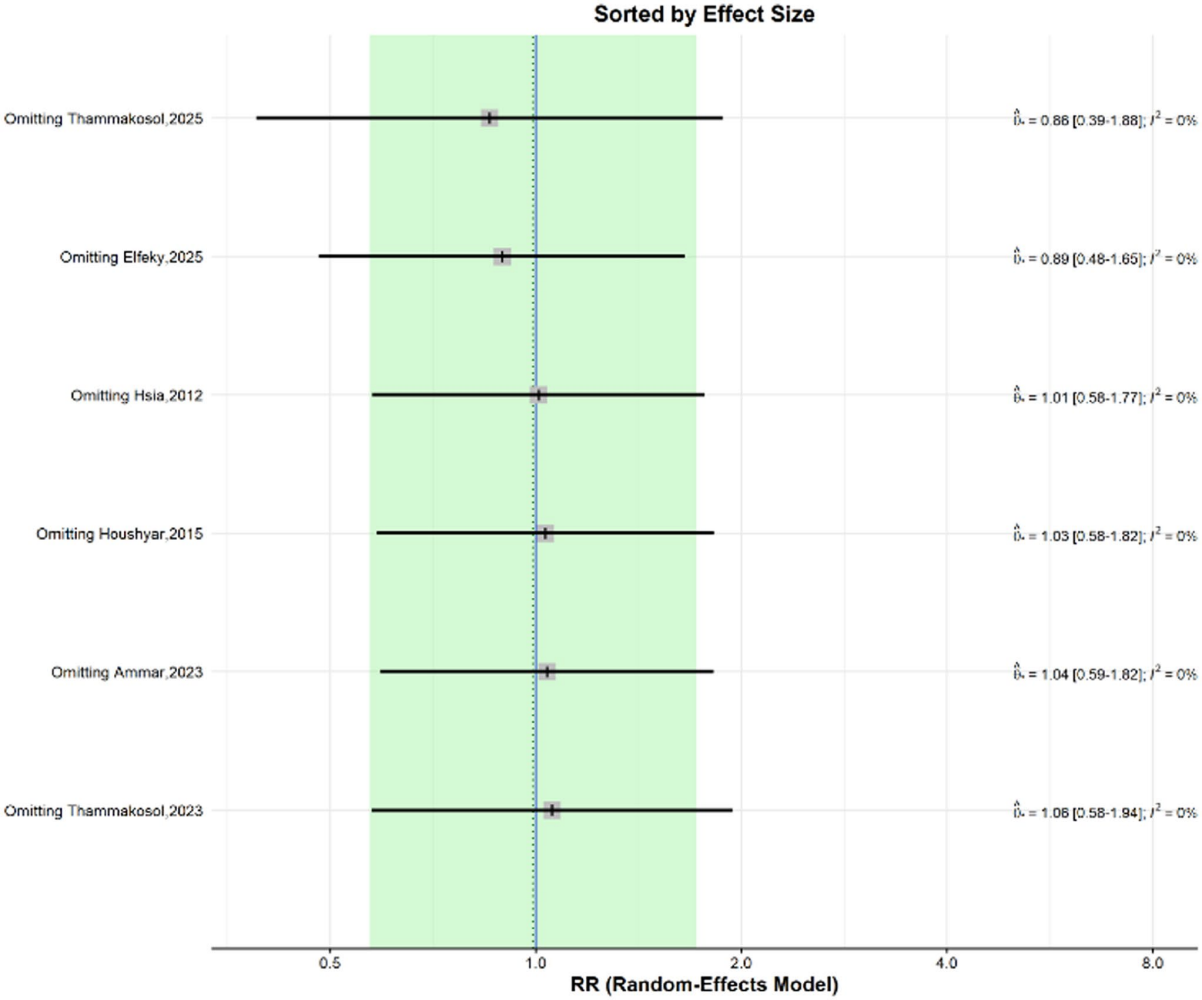
Forest plot of sensitivity analysis (leave‐one‐out) for rebound hyperglycaemia.

The rebound hyperglycaemia was reported in six studies. Although the pooled random‐effect meta‐analysis showed a basal insulin decrease, the rebound hyperglycaemia with RR 0.99 (95% CI: 0.57–1.72; *p* = 0.099), but with no significant difference between groups. Heterogeneity among studies was negligible, with an *I* ^2^ of 0% and a non‐significant Q‐test (*p* = 0.84), suggesting that the results were consistent across studies (Figure 8). Sensitivity analysis was conducted after removing the Hasia 2012 study, which lowered the heterogeneity for (*I* ^2^ 19.8%; Figure 9). The total effect size becomes RR 0.96, but still not significant. The meta‐regression analysis did not find any significant moderator effects. None of the covariates (male proportion, age, BMI, or sample size) was significantly associated with rebound hyperglycaemia (all *p* > 0.05). The adjusted estimates remained similar to the unadjusted estimate (QM = 2.96, *p* = 0.23; Figure S8) The certainty of evidence was generally moderate due to concerns of risk of bias (Table S1).

#### 3.3.5 Safety Outcomes

For recurrent DKA, the pooled fixed effect meta‐analysis yielded a pooled risk ratio of 0.47 (95% CI 0.08 to 2.89, *p* = 0.42), although this finding did not reach statistical significance indicating no statistically significant reduction in recurrent DKA compared to controls. Heterogeneity was minimal (*I* ^2^ = 0%). The certainty of evidence was generally moderate due to concerns of risk of bias (Table S2). Our analysis revealed no statistically significant differences between basal insulin and standard care groups with respect to hypokalaemia, hypoglycaemia, or mortality. (Figures S1–S4). The meta‐regression models for safety outcomes did not find any significant moderator effects. None of the covariates (male proportion, age, BMI, or sample size) was significantly associated with safety outcomes (all *p* > 0.05; Figures S9–S12).

## 4 Discussion

In this systematic review and meta‐analysis, we studied the use of early basal insulin while patients are on IV insulin infusion (IVII) in the acute phase of DKA. We demonstrated that the addition of basal insulin reduced the total amount of fluid and DKA resolution time significantly without increasing the risk of hypoglycaemia or hypokalaemia when compared to standard care alone. However, no significant difference was observed in length of stay, recurrent episodes of DKA and rebound hypoglycaemia between the two groups.

There were many RCTs that showed using early basal insulin (glargine, degludec, NPH) during the IV insulin therapy shortens DKA resolution and often reduces the total amount of insulin. A potential explanation for this is long‐acting insulin such as Degludec, a peakless agent that lasts up to 42 h, which provides steady basal insulin coverage, suppresses ketogenesis and helps maintain glucose control after transitioning from IVII, making it a great option for early incorporation with standard of care in treatment of DKA [10, 24]. The moderate heterogeneity observed in the primary outcome may reflect variability in intervention timing, patient characteristics and clinical protocols. Notably, sensitivity analysis demonstrated consistent findings after exclusion of individual studies, supporting the robustness of the overall effect. A meta‐analysis published in 2026, including eight RCTs, confirmed significantly faster DKA resolution and reduced IV insulin requirements with early SC basal insulin and importantly, no increase in adverse events, including hypoglycaemia and hypokalaemia, compared to standard care alone [25]. The steady state achieved by long‐acting insulin will suppress lipolysis and ketogenesis, while the ultra‐short‐acting insulin would target rapid correction of hyperglycaemia. The significant associations of total insulin dose with age, sex, BMI and study size in our meta‐regression suggest that individual patient characteristics may influence insulin requirements during DKA management, highlighting the importance of individualised treatment protocols.

For reducing the total amount of fluid with early basal insulin, with a mean decrease of around 400 mL compared to standard care. Although its modest amount of fluid, it's biologically plausible: when there is more efficient ketogenesis suppression, osmotic diuresis should lessen ongoing fluid loss, especially if DKA resolution is accelerated. However, it's important to mention that the previous DKA fluid trials exhibited substantially larger differences in fluid volume or infusion rates, often resulting in minimal or inconsistent effects on clinical outcomes, suggesting that the observed reduction of approximately 400 mL may not translate into meaningful clinical benefit [26]. Usually, the clinical significance of fluid reduction is determined by multiple factors such as baseline dehydration severity, comorbid conditions and institutional protocols [27]. Furthermore, variability in fluid resuscitation protocols across studies reflects ongoing clinical uncertainty regarding optimal fluid type and administration rates in DKA, an area that continues to evolve in recent literature.

In addition, our meta‐analysis could not find a statistical significance for the reduction in the LOS. The observed reduction in hospital stay, although not statistically significant, may still be clinically relevant, particularly in resource‐limited settings. The wide confidence interval suggests insufficient power rather than the absence of effect. Although the findings do not exclude a clinically important reduction in LOS, the precision of the estimated effect remains limited and the true magnitude of benefit is uncertain. In addition, the LOS multifactorial outcome is influenced not only by metabolic resolution but also by healthcare system practices, discharge planning and patient‐specific factors, which may attenuate the measurable impact of insulin strategies alone [28, 29].

Other secondary outcomes studied were rebound hyperglycaemia, which was reported as an outcome across six of the eight included studies, and no significant statistical difference was found between the intervention and control groups. This reflects the well‐known fact that multiple precipitating factors, such as infection and steroid use, can alter inpatient glycaemic values, often resulting in adjustments according to individual patients' insulin requirements [30]. There was some heterogeneity (*I* ^2^ = 35.4%) noted, which can be explained by the discrepancy seen in the outcome reported by Hsia et al. [8]. This study reported a reduced incidence of rebound hyperglycaemia in the intervention group; however, it included both patients with DKA and postoperative patients with non‐DKA‐related hyperglycaemia [8]. A systematic review and meta‐analysis hypothesised that the non‐DKA hyperglycaemia patients in the intervention group may have experienced less stress with better glycaemic values, resulting in lower incidences of rebound hyperglycaemia [22]. Recurrence of DKA was studied as an outcome across three studies and although the analyses showed that early initiation reduced the risk of recurrence of hypoglycaemia, this finding was not statistically significant.

The absence of significant differences in recurrent DKA, hypoglycaemia, hypokalaemia and mortality between groups supports the safety of early SC basal insulin. Consistent negligible heterogeneity for these outcomes further strengthens this interpretation. Interestingly, while earlier reviews and longstanding clinical tradition have limited SC insulin to the post‐resolution phase of DKA, the current guidelines are progressively acknowledging the viability of early basal insulin co‐administration, particularly in the case of mild to moderate DKA [25].

The absence of significant differences in recurrent DKA after discharge suggests that early basal insulin does not adversely influence metabolic stability in the short term. However, data on long‐term DKA recurrence and post‐discharge readmission are scarce in the current literature and future research should address these clinically relevant endpoints. Early basal insulin appears safe within the studied populations; however, further research is needed to confirm safety in higher‐risk groups.

## 5 Limitations

There are several limitations present in the current meta‐analysis. Firstly, we included open‐label designed RCTs in our analyses, which introduced observer bias as the clinicians were not blinded to treatment allocation. Second, the studies were based on single‐centre facilities, testing the efficacy of early introduction of basal insulin in DKA on a small subset of the population. Lastly, patients with advanced kidney disease, euglycaemic DKA (plasma glucose < 200–250 mg/dL) were excluded from the included studies, which could limit the generalisability of the above findings. Finally, the generalisability of our findings may be limited, as the included studies predominantly involved younger patients with moderate DKA. Data on elderly populations, severe DKA and patients with significant comorbidities remain limited.

## 6 Future Directions

Future research should focus on large, multicentre RCTs that standardise insulin protocols, clearly define outcomes such as LOS and long‐term recurrence, and explore patient‐centred outcomes, including quality of life and healthcare resource utilisation. The potential role of patient characteristics such as BMI, baseline insulin requirements and age in modulating response to basal insulin during DKA warrants further mechanistic and clinical investigation. In addition, longer‐term follow‐up studies assessing post‐hospital metabolic control and readmissions will help clarify the broader impact of early basal insulin administration.

## 7 Conclusion

Our findings support the inclusion of early SC basal insulin in DKA management protocols as a strategy to optimise insulin delivery and potentially decrease resource utilisation. The early addition of basal insulin may facilitate a smoother transition from IVII to maintenance regimens, potentially reducing metabolic rebound and improving workflow efficiencies in acute care settings. While traditional protocols have delayed basal insulin until imminent transition off IVII to avoid overlapping effects, emerging evidence suggests that early basal dosing can be safe and effective, particularly with appropriate monitoring.

## Author Contributions

**Insiya Mohammed Rampurawala:** data curation, investigation. **Maha AbuZarifa:** writing – review and editing, data curation, validation, visualization, software, formal analysis. **Rejean R. R. Sawh:** resources. **Muhammad Bin Salman:** writing – original draft. **Abdallah AbuJlambo:** conceptualization, methodology, formal analysis, writing – original draft, software, validation, visualization, project administration. **Yara Ashour:** investigation, data curation. **Subhash Reddy Narayana Reddy N:** data curation, investigation. **Hazem Ayesh:** supervision, validation, visualization, formal analysis, software.

## Funding

The authors have nothing to report.

## Ethics Statement

This study was conducted using publicly available studies and so the Institutional Review Board approval and informed consent were not required.

## Consent

The authors have nothing to report.

## Conflicts of Interest

The authors declare no conflicts of interest.

## Supporting information


**Figure S1:** Forest plot for risk of diabetic ketoacidosis (DKA) recurrence comparing early basal insulin versus standard care.
**Figure S2:** Forest plot for risk of hypokalaemia comparing early basal insulin versus standard care.
**Figure S3:** Forest plot for risk of hypoglycaemia comparing early basal insulin versus standard care.
**Figure S4:** Forest plot for in‐hospital mortality comparing early basal insulin versus standard care in patients with diabetic ketoacidosis.
**Figure S5:** Meta‐regression analysis of time to DKA resolution according to mean age, BMI, sample size and proportion of male participants.
**Figure S6:** Meta‐regression analysis of total intravenous fluid volume according to mean age, BMI, sample size and proportion of male participants.
**Figure S7:** Meta‐regression analysis of length of hospital stay according to mean age, BMI, sample size and proportion of male participants.
**Figure S8:** Meta‐regression analysis of rebound hyperglycaemia according to mean age, BMI, sample size and proportion of male participants.
**Figure S9:** Meta‐regression analysis of recurrent DKA according to mean age, BMI, sample size and proportion of male participants.
**Figure S10:** Meta‐regression analysis of hypokalaemia according to mean age, BMI, sample size and proportion of male participants.
**Figure S11:** Meta‐regression analysis of hypoglycaemia according to mean age, BMI, sample size and proportion of male participants.
**Figure S12:** Meta‐regression analysis of in‐hospital mortality according to mean age, BMI, sample size and proportion of male participants.
**Table S1:** Summary of certainty of evidence using the Grading of Recommendations Assessment, Development and Evaluation (GRADE) approach for all outcomes.
**Table S2:** Detailed GRADE assessment of included outcomes, including risk of bias, inconsistency, indirectness, imprecision and publication bias.

## Data Availability

The data that support the findings of this study are available from the corresponding author upon reasonable request.
