# Supplementary material for: Impact of Early Subcutaneous Basal Insulin With Intravenous Insulin Infusion for Diabetic Ketoacidosis and Glycaemic Outcomes: A Systematic Review and Meta‐Analysis
Source: Endocrinol Diabetes Metab. 2026 Jul 6;9(4):e70277. doi: 10.1002/edm2.70277 (PMC13334457; doi:10.1002/edm2.70277)
Supplement: Supplementary file 1 — Figure S1: Forest plot for risk of diabetic ketoacidosis (DKA) recurrence comparing early basal insulin versus standard care. Figure S2: Forest plot for risk of hypokalaemia comparing early basal insulin versus standard care. Figure S3: Forest plot for risk of hypoglycaemia comparing early basal insulin versus standard care. Figure S4: Forest plot for in‐hospital mortality comparing early basal insulin versus standard care in patients with diabetic ketoacidosis. Figure S5: Meta‐regression analysis of time to DKA resolution according to mean age, BMI, sample size and proportion of male participants. Figure S6: Meta‐regression analysis of total intravenous fluid volume according to mean age, BMI, sample size and proportion of male participants. Figure S7: Meta‐regression analysis of length of hospital stay according to mean age, BMI, sample size and proportion of male participants. Figure S8: Meta‐regression analysis of rebound hyperglycaemia according to mean age, BMI, sample size and proportion of male participants. Figure S9: Meta‐regression analysis of recurrent DKA according to mean age, BMI, sample size and proportion of male participants. Figure S10: Meta‐regression analysis of hypokalaemia according to mean age, BMI, sample size and proportion of male participants. Figure S11: Meta‐regression analysis of hypoglycaemia according to mean age, BMI, sample size and proportion of male participants. Figure S12: Meta‐regression analysis of in‐hospital mortality according to mean age, BMI, sample size and proportion of male participants. Table S1: Summary of certainty of evidence using the Grading of Recommendations Assessment, Development and Evaluation (GRADE) approach for all outcomes. Table S2: Detailed GRADE assessment of included outcomes, including risk of bias, inconsistency, indirectness, imprecision and publication bias. [file EDM2-9-e70277-s001.docx]

**
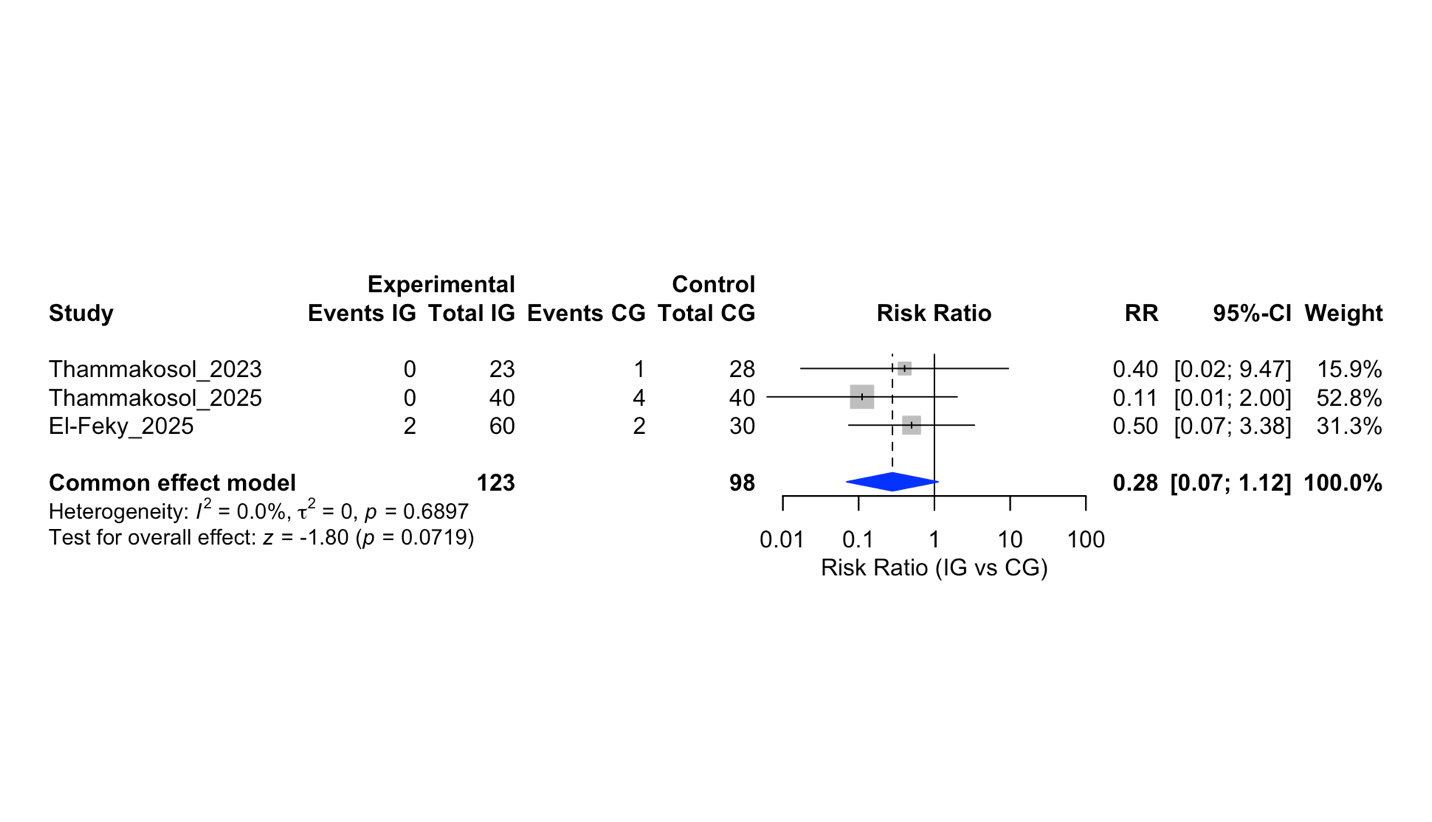
**

**Supplementary Figure 1:** Forest plot for risk of diabetic ketoacidosis (DKA) recurrence comparing early basal insulin versus standard care.

**
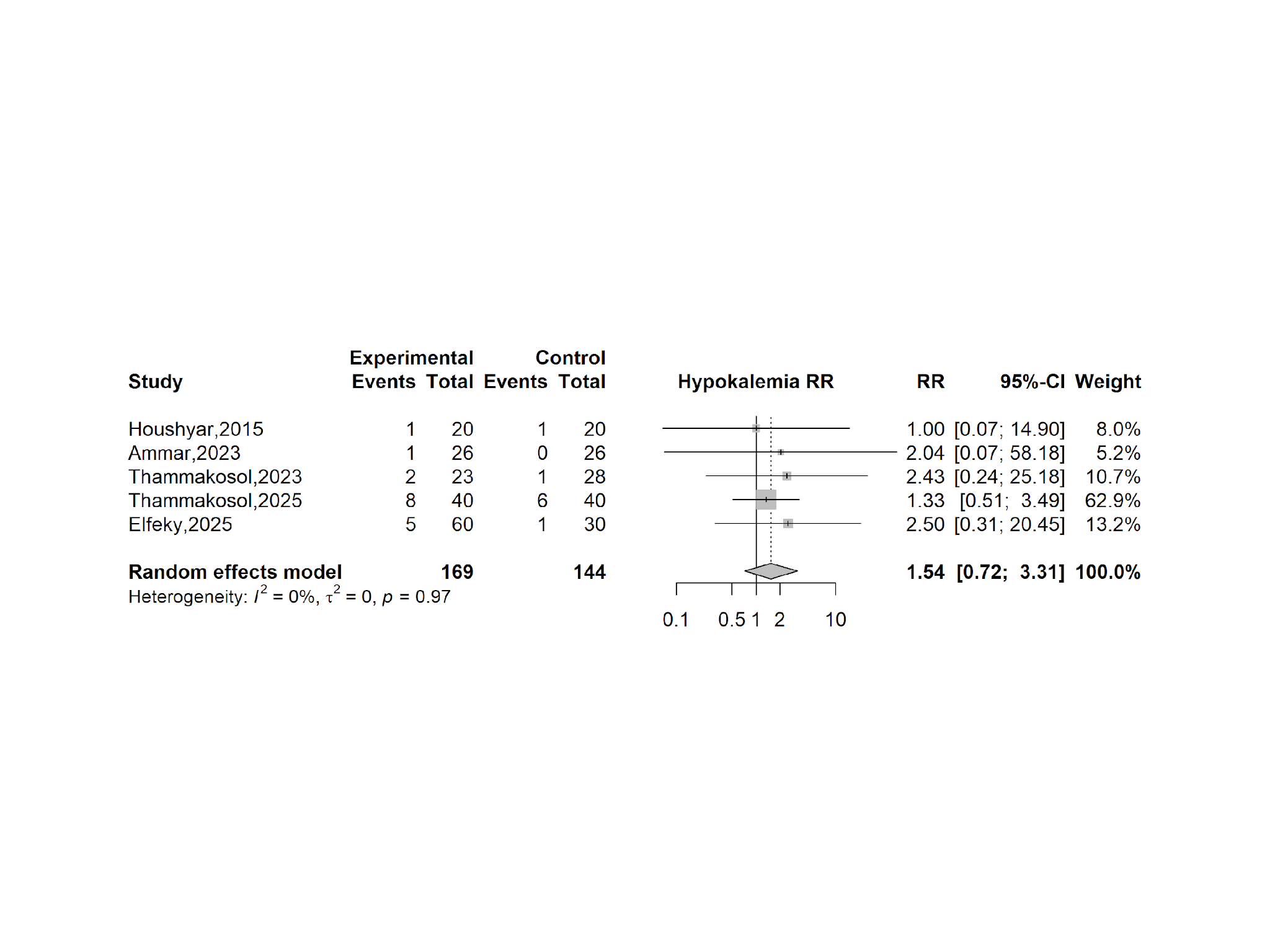
**

**Supplementary Figure 2**: Forest plot for risk of hypokalemia comparing early basal insulin versus standard care.


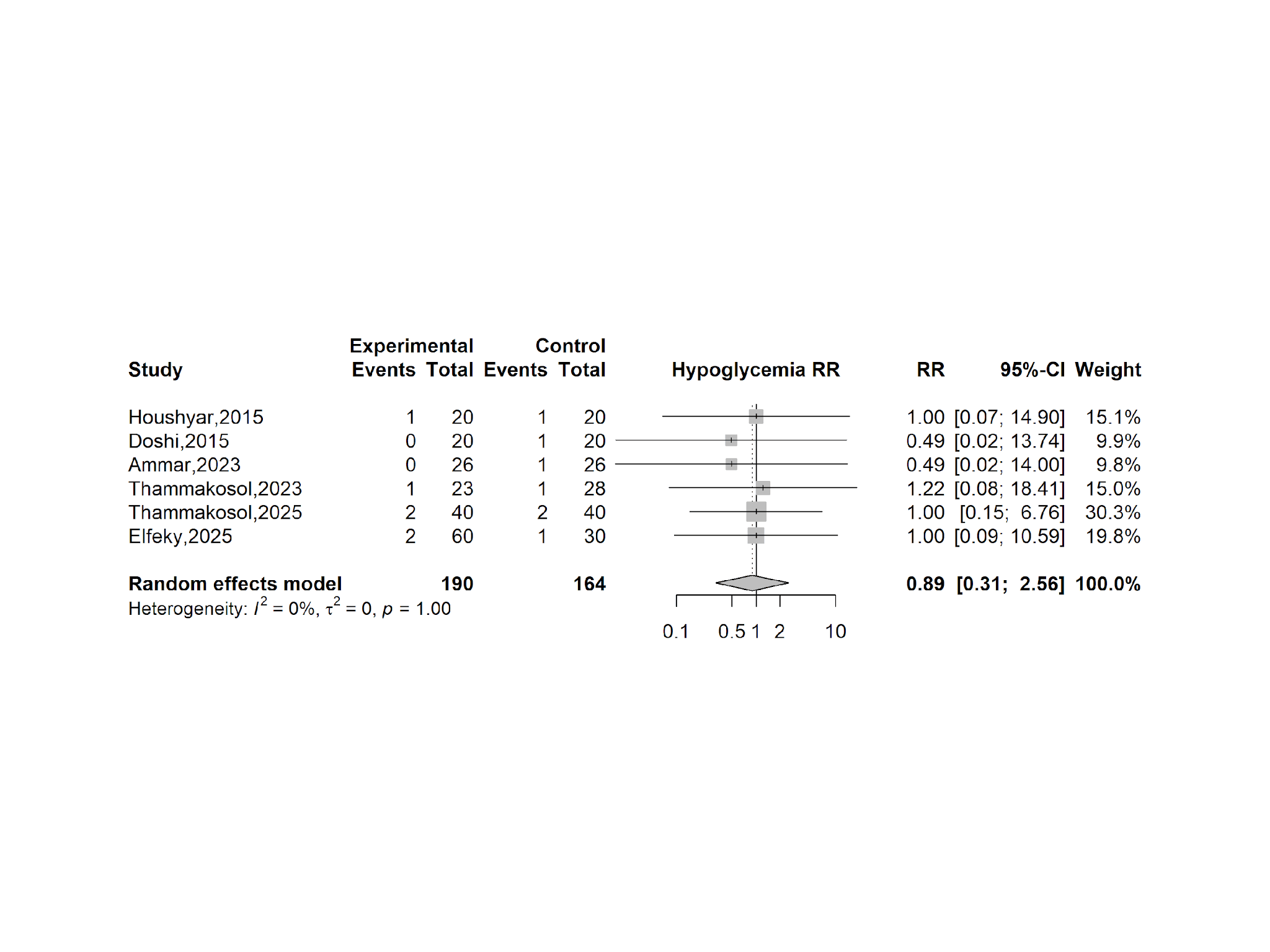


**Supplementary Figure 3:** Forest plot for risk of hypoglycemia comparing early basal insulin versus standard care.

**
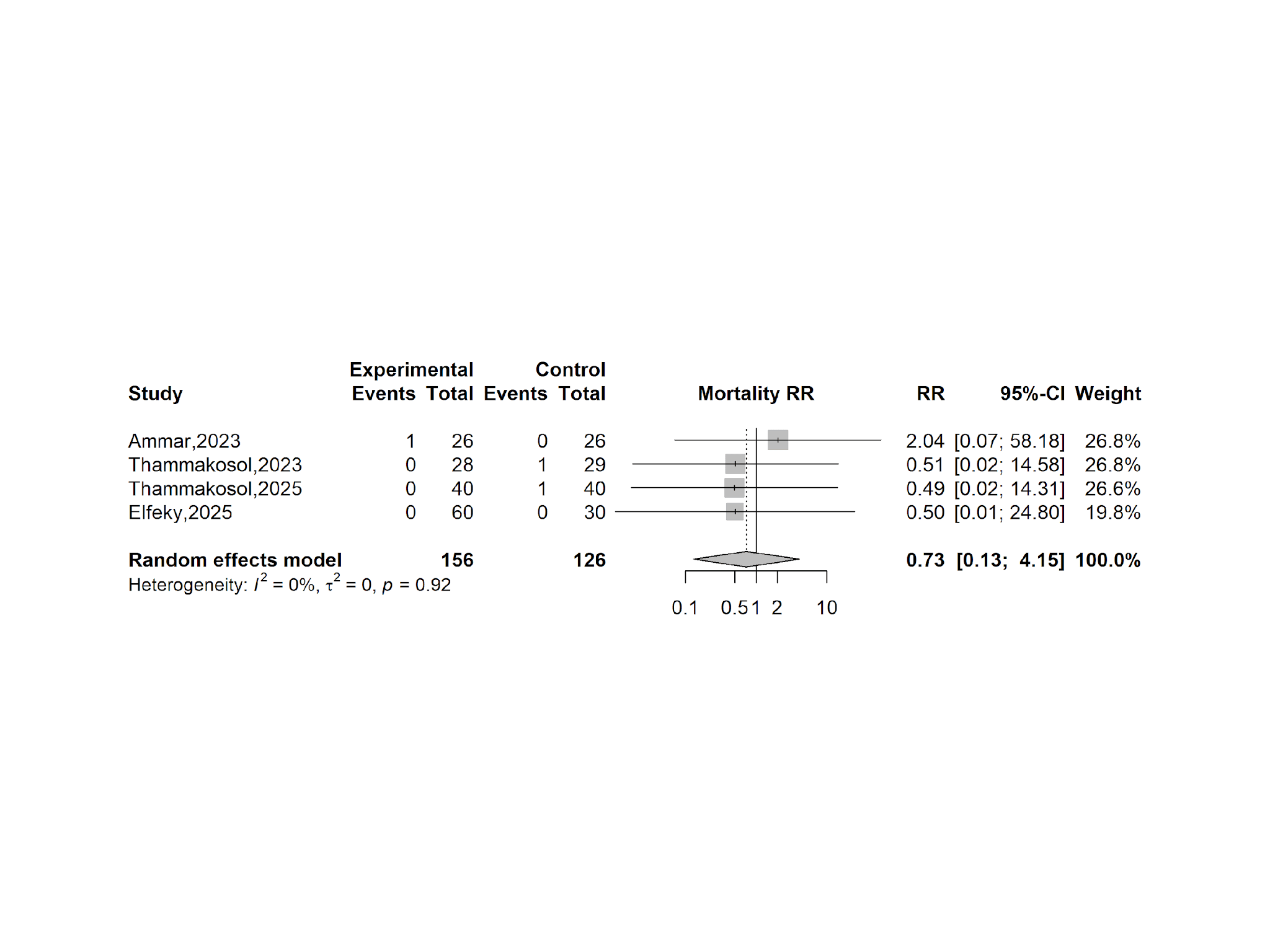
**

**Supplementary Figure 4**: Forest plot for in-hospital mortality comparing early basal insulin versus standard care in patients with diabetic ketoacidosis.

| 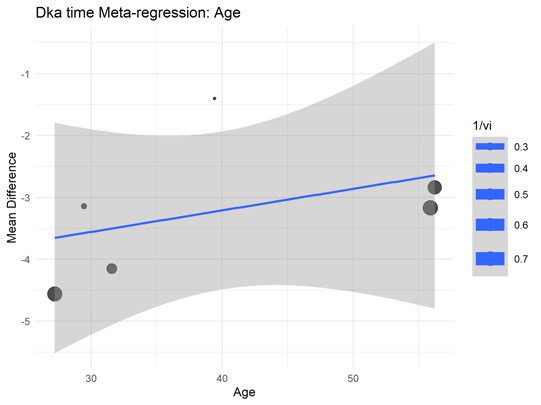 | 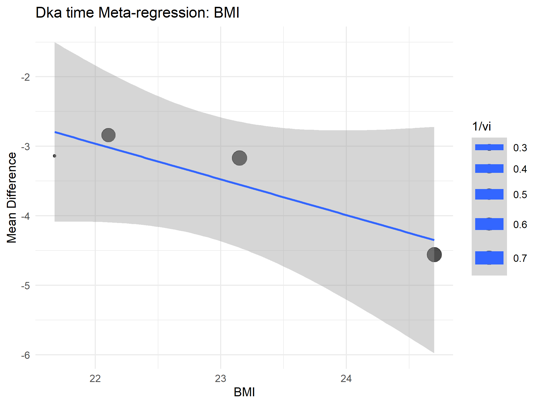 |
| --- | --- |
| 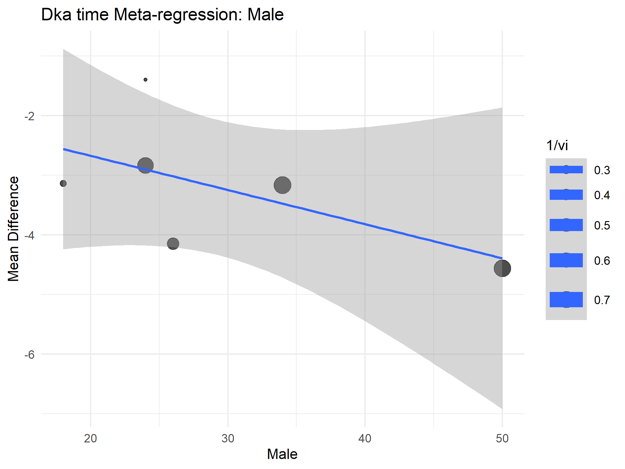 | 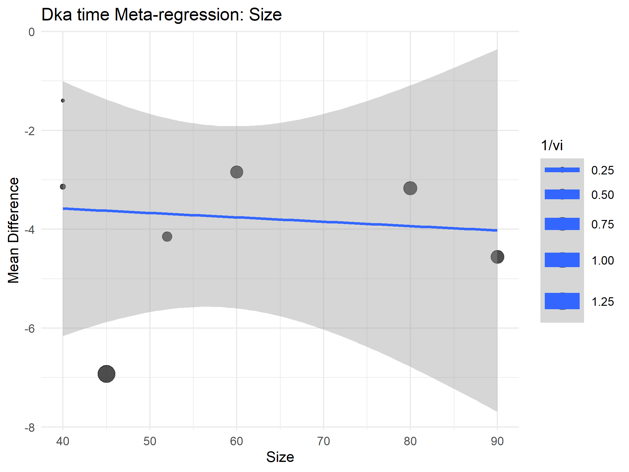 |

**Supplementary Figure 5:** Meta-regression analysis of time for DKA resolution according to mean age, BMI, sample size, and proportion of male participants.

| 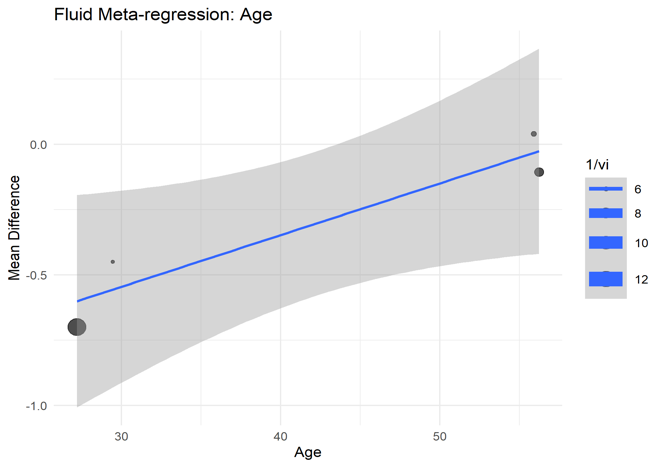 | 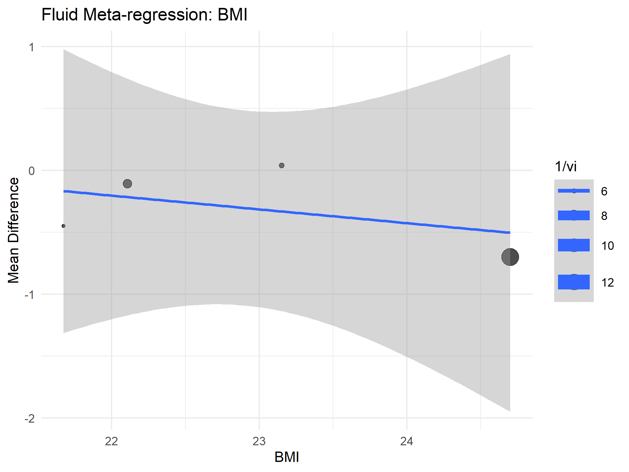 |
| --- | --- |
| 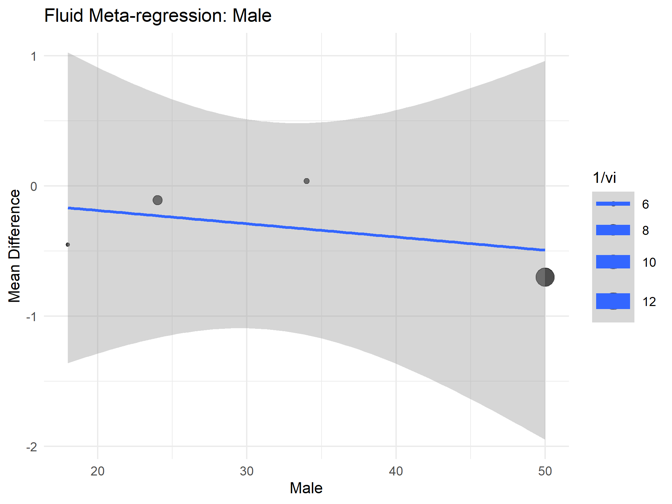 | 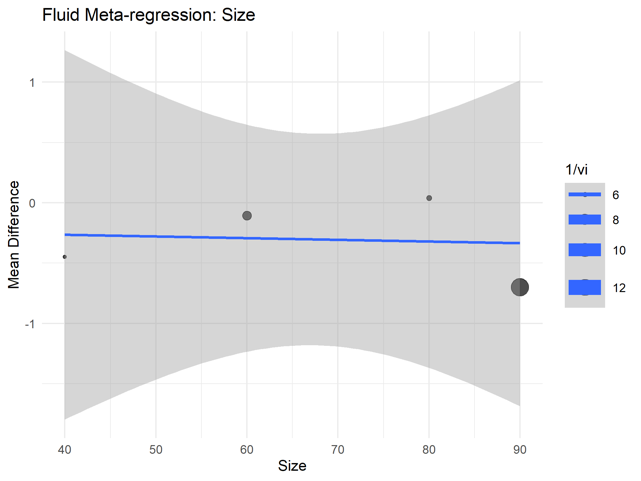 |

**Supplementary Figure 6:** Meta-regression analysis of total intravenous fluid volume according to mean age, BMI, sample size, and proportion of male participants.

| **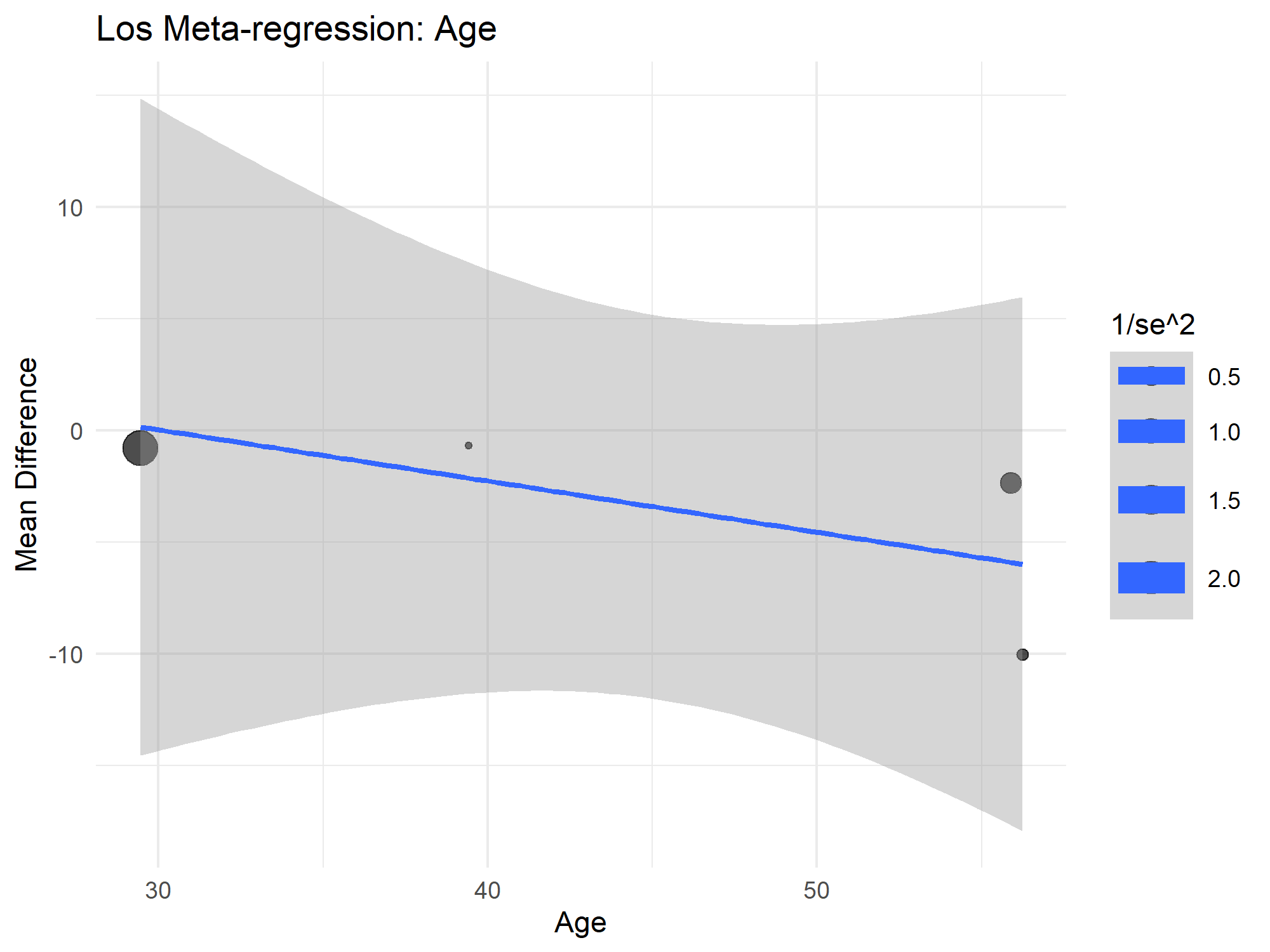** | **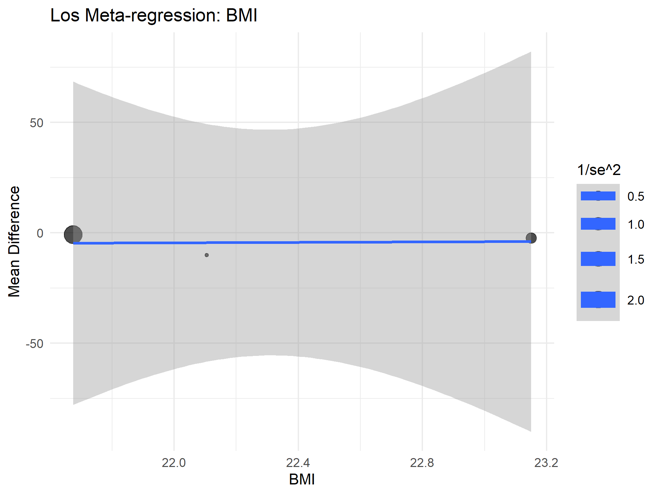** |
| --- | --- |
| **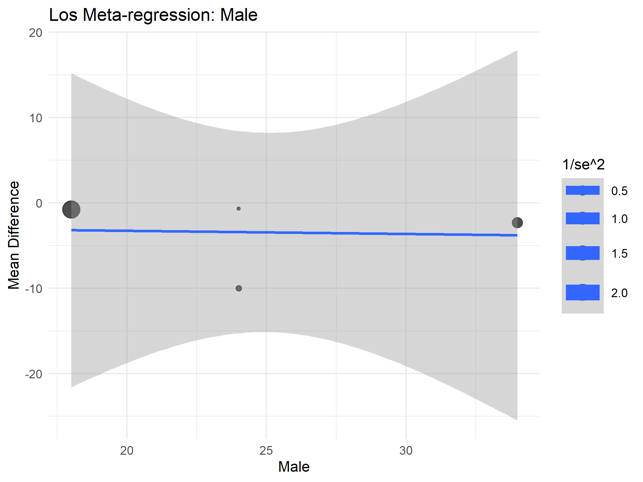** | **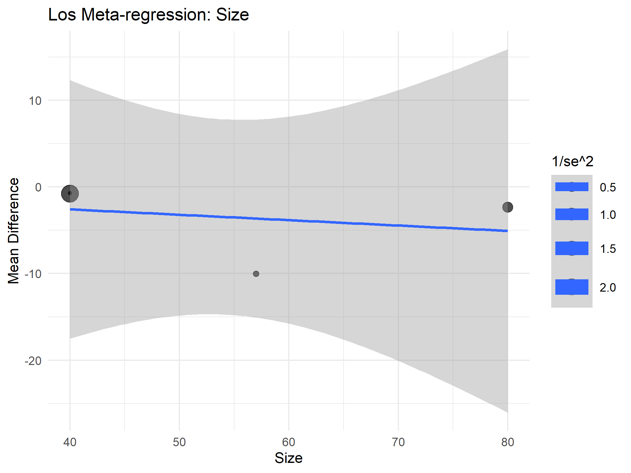** |

**Supplementary Figure 7:** Meta-regression analysis of length of hospital stay according to mean age, BMI, sample size, and proportion of male participants.

| 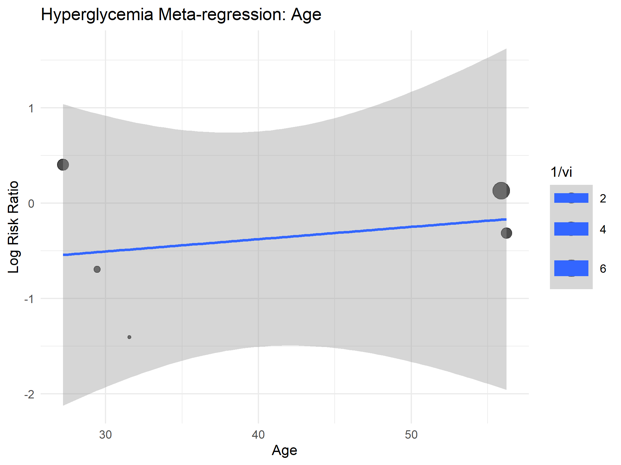 | 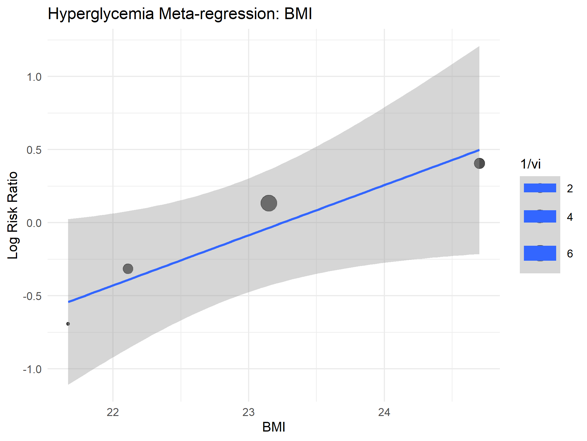 |
| --- | --- |
| 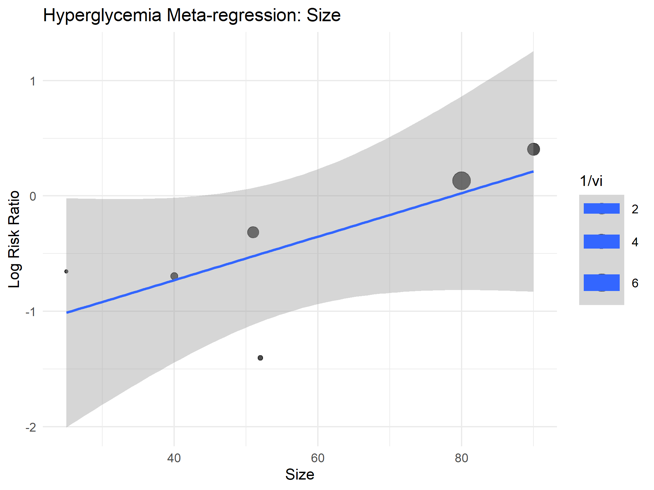 | 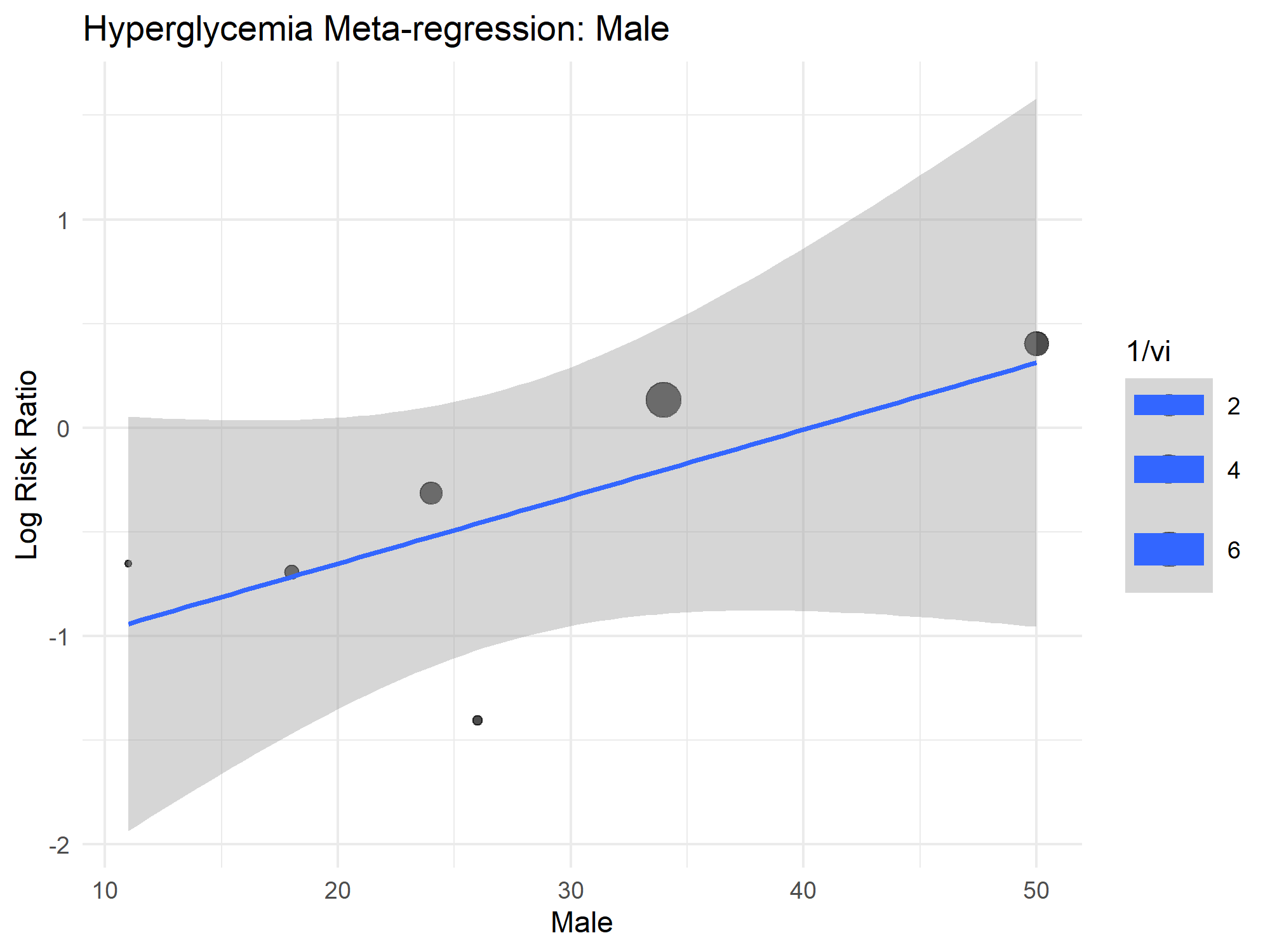 |

**Supplementary Figure 8:** Meta-regression analysis of rebound hyperglycemia according to mean age, BMI, sample size, and proportion of male participants.

| 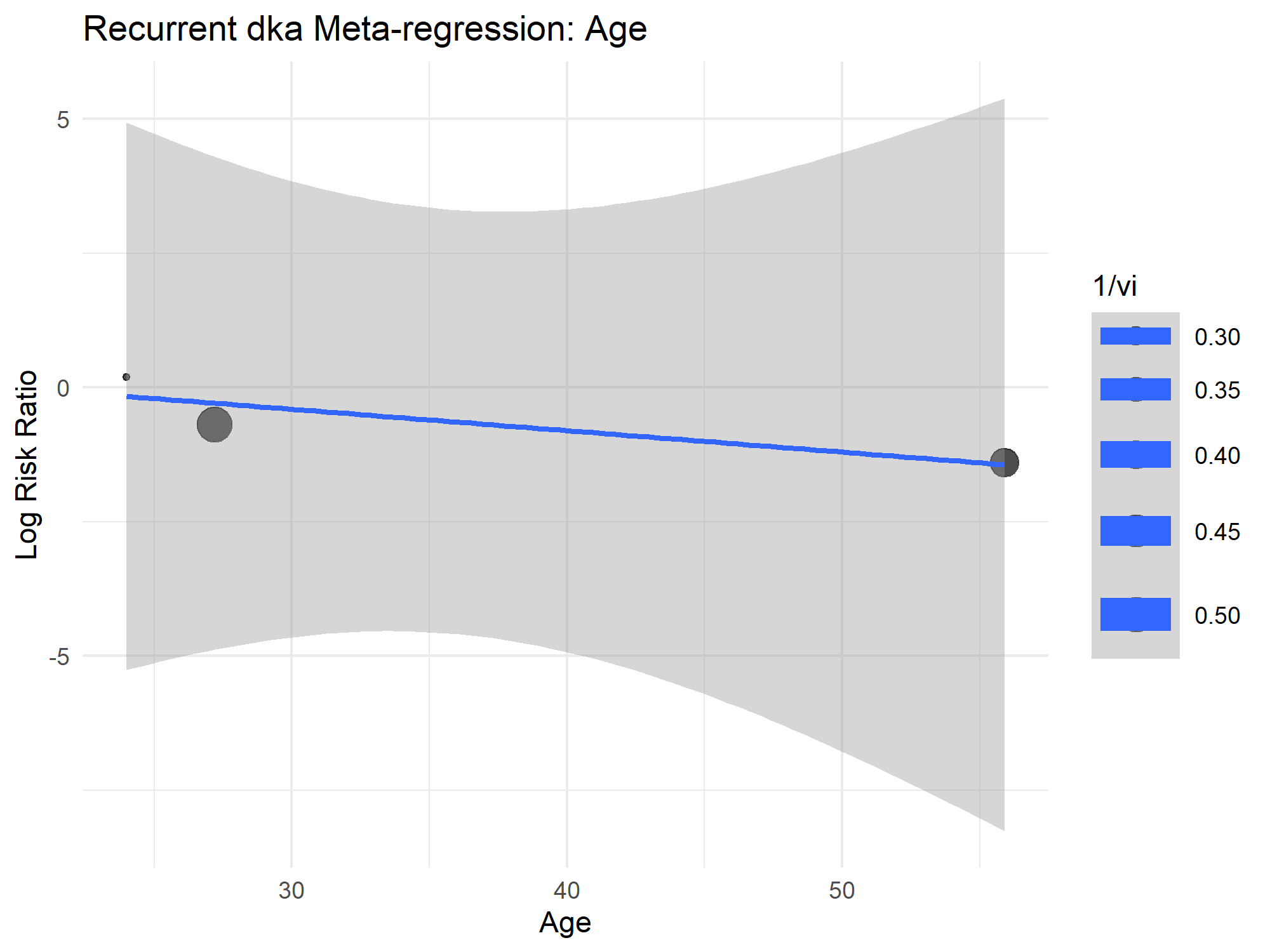 | 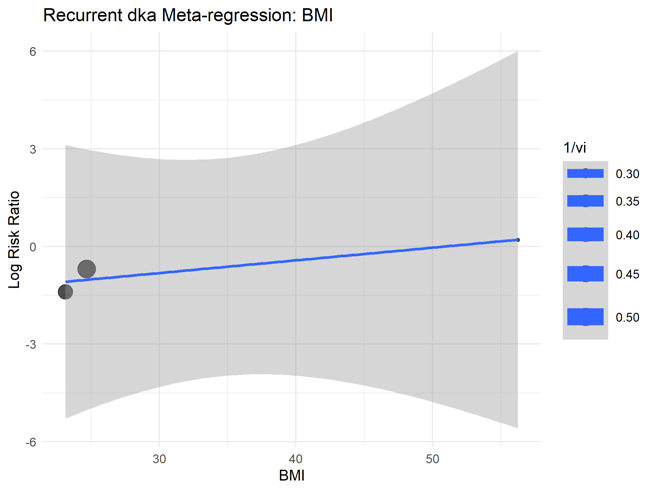 |
| --- | --- |
| 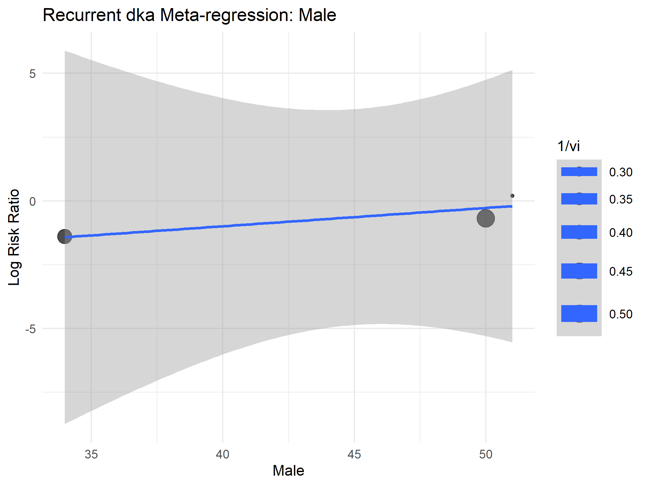 |  |

**Supplementary Figure 9:** Meta-regression analysis of recurrent DKA according to mean age, BMI, sample size, and proportion of male participants.

| 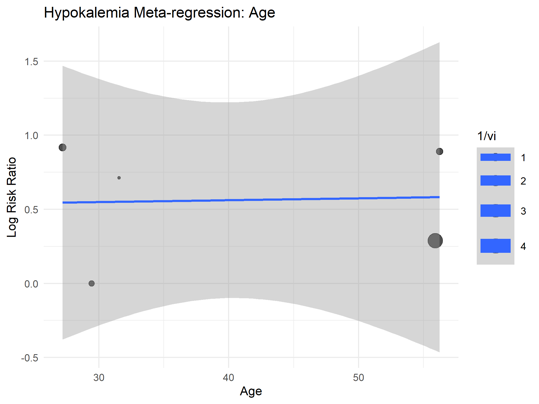 | 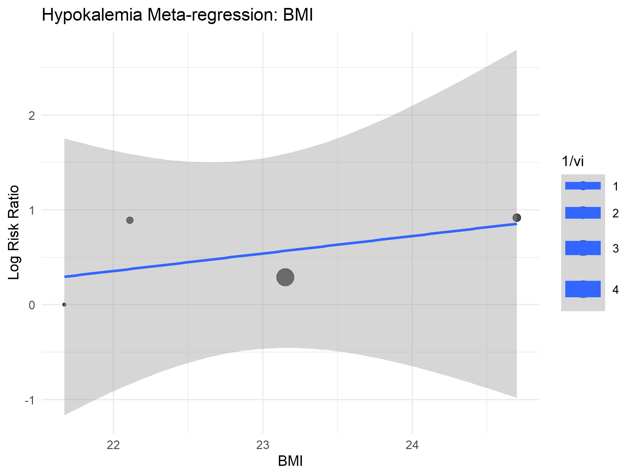 |
| --- | --- |
| 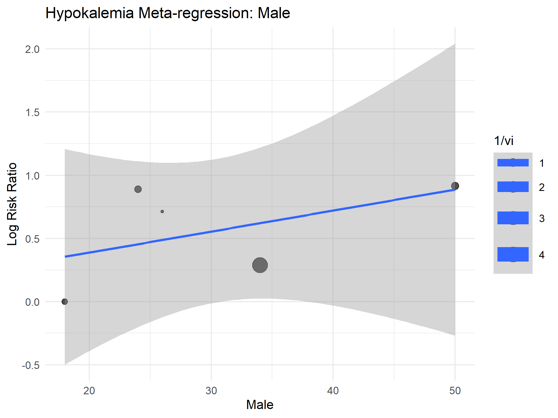 | 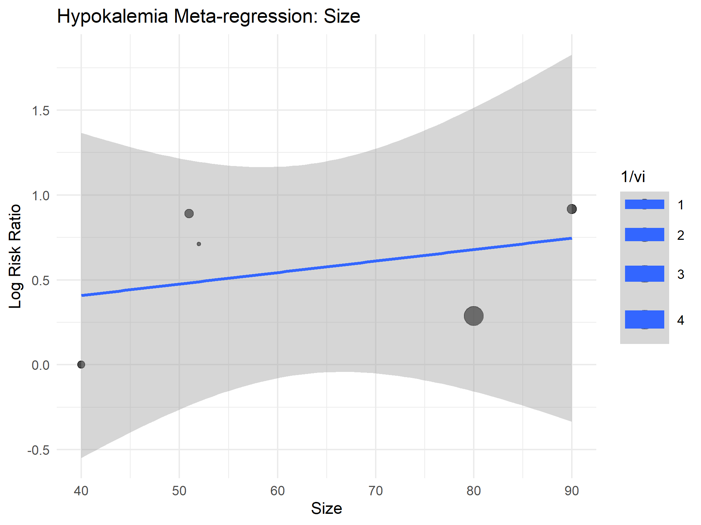 |

**Supplementary Figure 10:** Meta-regression analysis of hypokalemia according to mean age, BMI, sample size, and proportion of male participants.

| 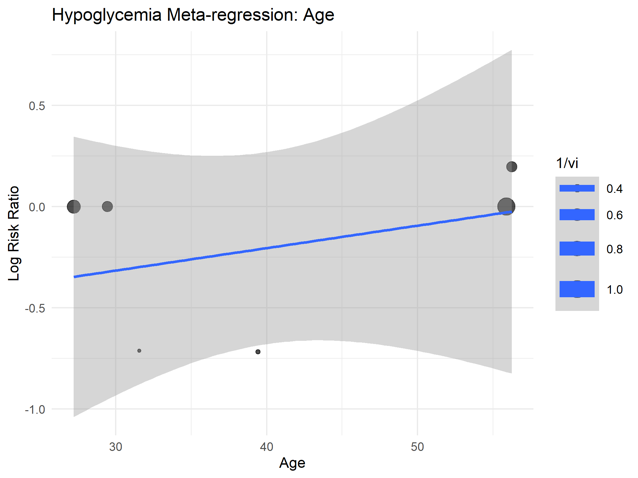 | 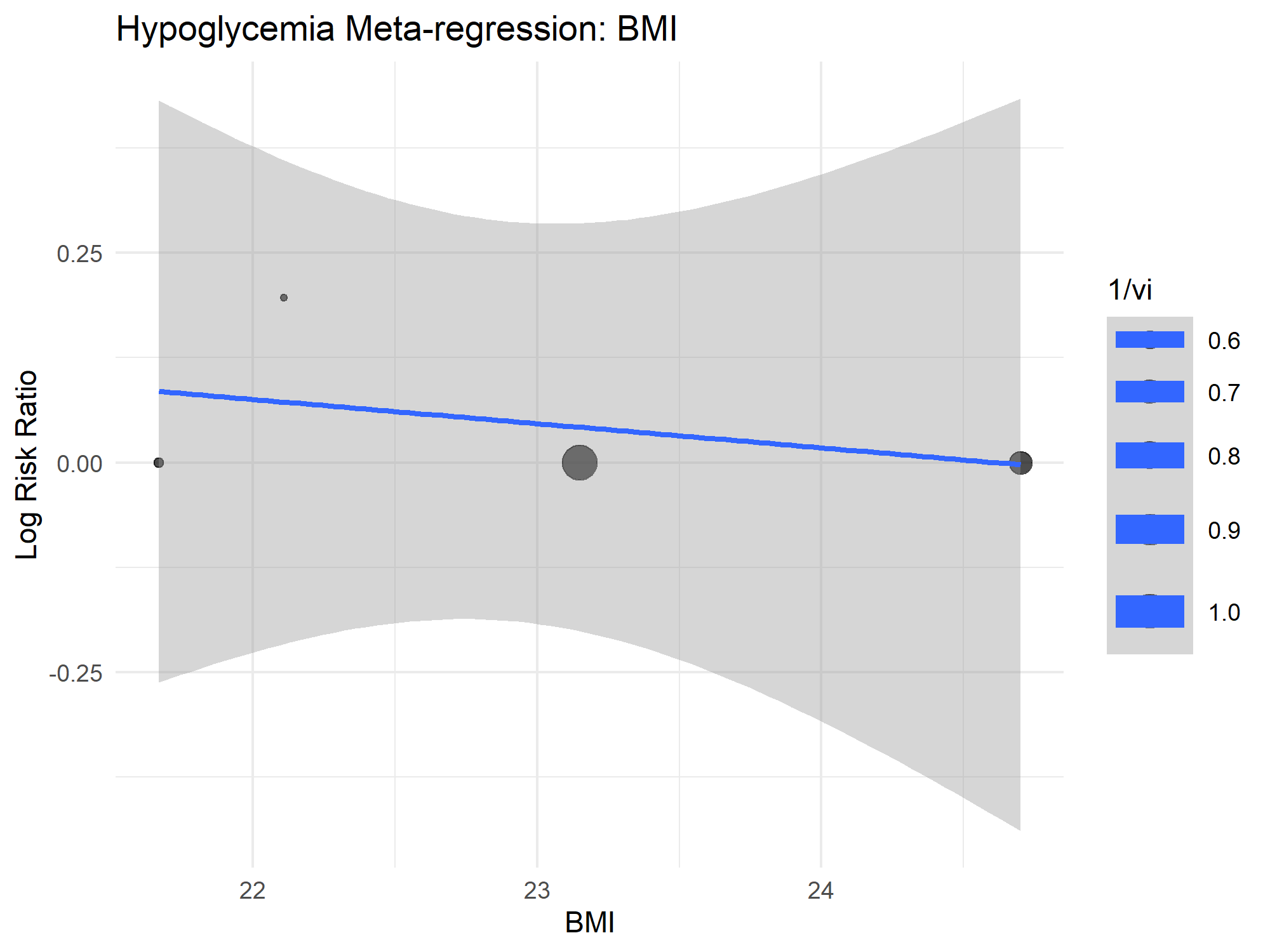 |
| --- | --- |
| 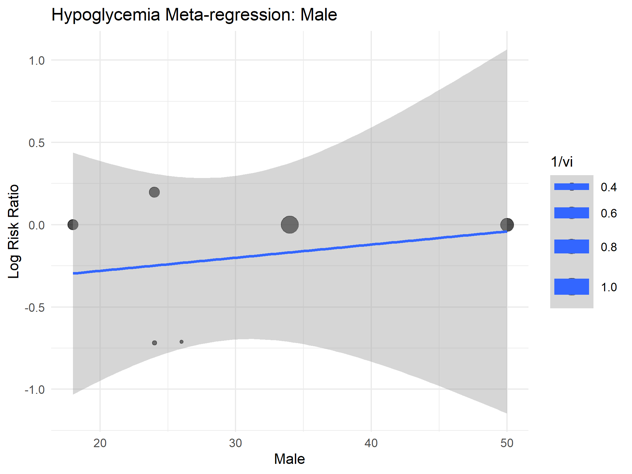 | 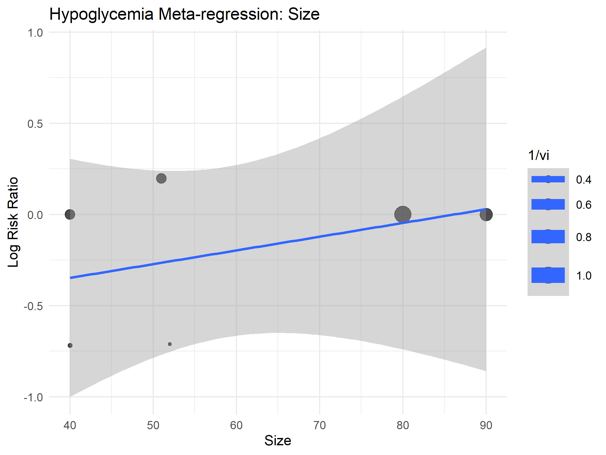 |

**Supplementary Figure 11:** Meta-regression analysis of hypoglycemia according to mean age, BMI, sample size, and proportion of male participants.

| 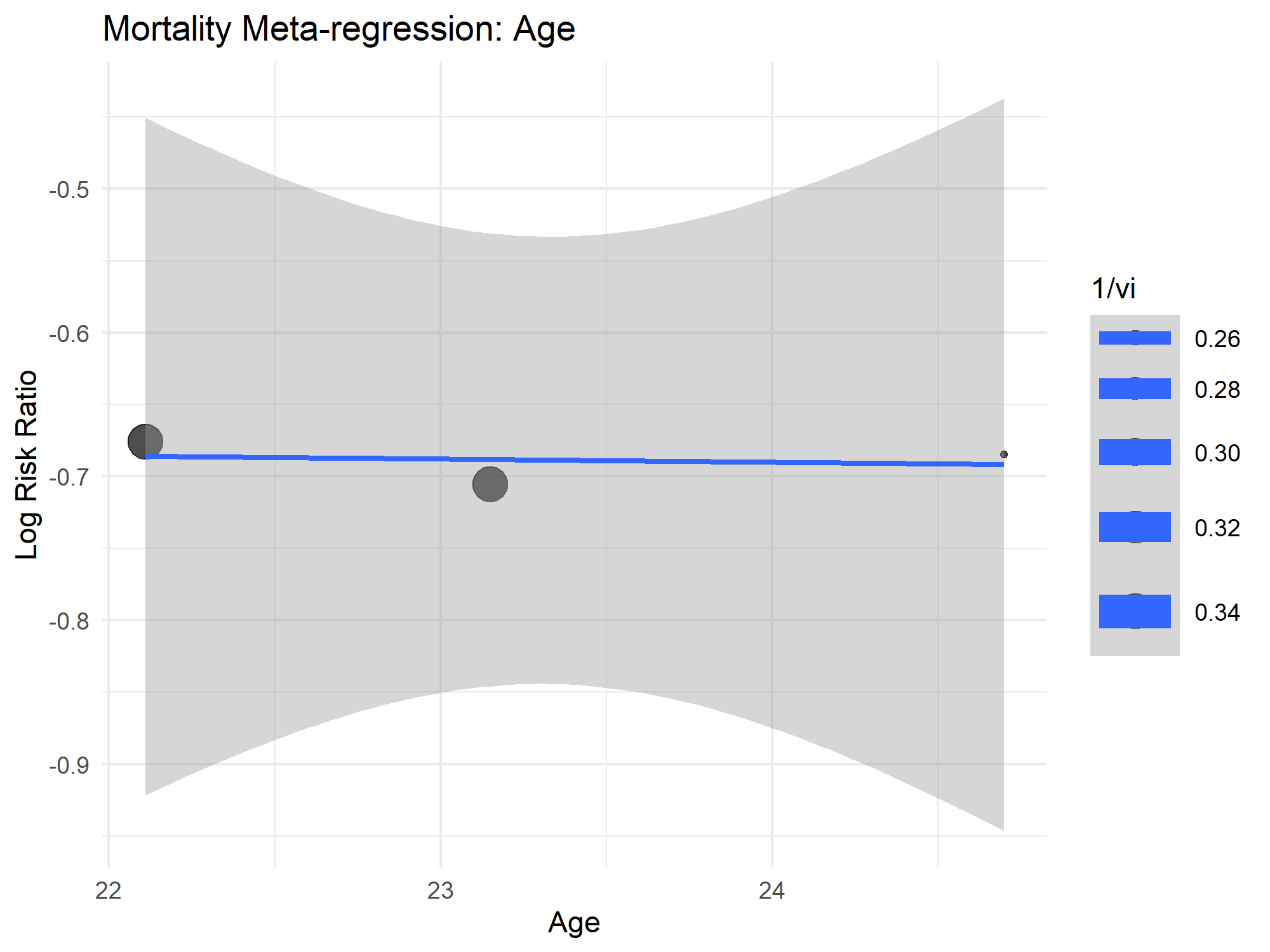 | 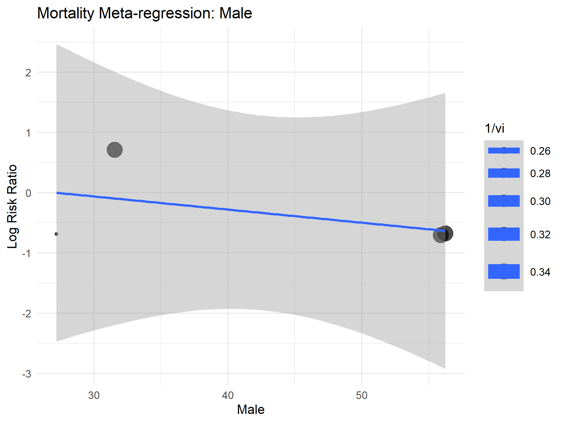 |
| --- | --- |
| 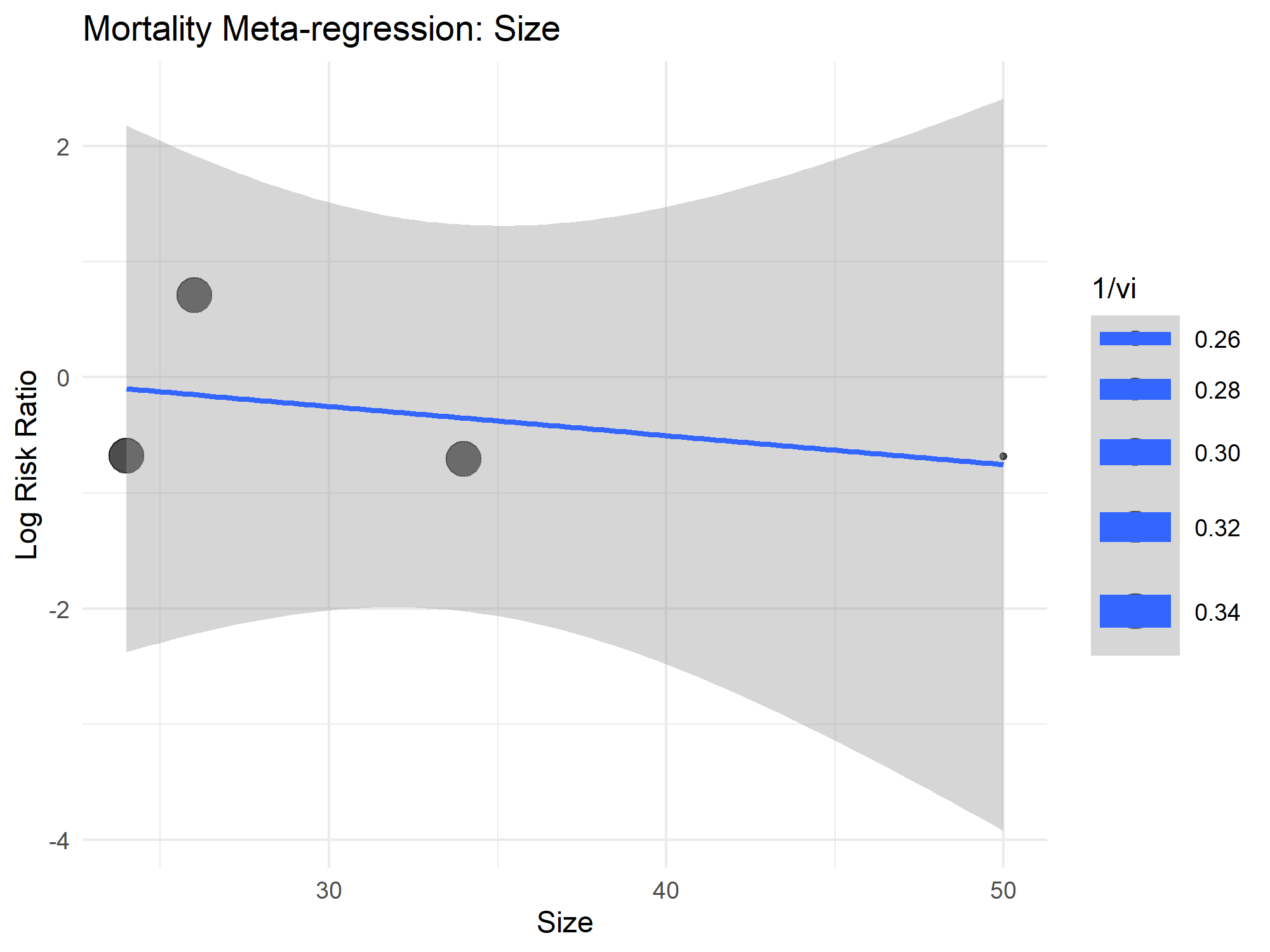 |  |

**Supplementary Figure 12:** Meta-regression analysis of in-hospital mortality according to mean age, BMI, sample size, and proportion of male participants.

| **Summary of findings:** | | | | | | |
| --- | --- | --- | --- | --- | --- | --- |
| **Basal compared to SQ Insulin for diabetic ketoacidosis: a SRMA for DKA** | | | | | | |
| **Patient or population:** diabetic ketoacidosis: a SRMA for DKA  **Setting:**  **Intervention:** Basal  **Comparison:** SQ Insulin | | | | | | |
| Outcomes | **Anticipated absolute effects^*^** (95% CI) | | Relative effect (95% CI) | № of participants (studies) | Certainty of the evidence (GRADE) | Comments |
|  | **Risk with SQ Insulin** | **Risk with Basal** |  |  |  |  |
| Time to Resolution of DKA | The mean time to Resolution of DKA was **0** | MD **4.07 lower** (5.73 lower to 2.42 lower) | - | 407 (6 RCTs) | ⨁⨁⨁◯ Moderate^a^ |  |
| Length of Hospital Stay | The mean length of Hospital Stay was **0** | MD **2.38 lower** (7.94 lower to 3.17 higher) | - | 217 (3 RCTs) | ⨁⨁◯◯ Low^a,b^ |  |
| Rebound Hyperglycemia | 570 per 1,000 | **399 per 1,000** (610 to 262) | **RR 0.70** (0.46 to 1.07) | 367 (5 RCTs) | ⨁⨁⨁◯ Moderate^a^ |  |
| Recurrent DKA | 56 per 1,000 | **26 per 1,000** (92 to 8) | **RR 0.47** (0.14 to 1.64) | 279 (3 RCTs) | ⨁⨁⨁◯ Moderate^a^ |  |
| ***The risk in the intervention group** (and its 95% confidence interval) is based on the assumed risk in the comparison group and the **relative effect** of the intervention (and its 95% CI).  **CI:** confidence interval; **MD:** mean difference; **RR:** risk ratio | | | | | | |
| **GRADE Working Group grades of evidence** **High certainty:** we are very confident that the true effect lies close to that of the estimate of the effect. **Moderate certainty:** we are moderately confident in the effect estimate: the true effect is likely to be close to the estimate of the effect, but there is a possibility that it is substantially different. **Low certainty:** our confidence in the effect estimate is limited: the true effect may be substantially different from the estimate of the effect. **Very low certainty:** we have very little confidence in the effect estimate: the true effect is likely to be substantially different from the estimate of effect. | | | | | | |

**Supplementary Table 1.** Summary of certainty of evidence using the Grading of Recommendations Assessment, Development and Evaluation (GRADE) approach for all outcomes.

**Author(s):**

**Question:** Basal compared to SQ Insulin for diabetic ketoacidosis: a SRMA for DKA

**Setting:**

**Bibliography:** Early basal insulin administration during intravenous insulin infusion for diabetic ketoacidosis: a SRMA for DKA. Cochrane Database of Systematic Reviews [Year], Issue [Issue].

| **Certainty assessment** | | | | | | | **№ of patients** | | **Effect** | | **Certainty** | **Importance** |
| --- | --- | --- | --- | --- | --- | --- | --- | --- | --- | --- | --- | --- |
| **№ of studies** | **Study design** | **Risk of bias** | **Inconsistency** | **Indirectness** | **Imprecision** | **Other considerations** | **Basal** | **SQ Insulin** | **Relative (95% CI)** | **Absolute (95% CI)** |  |  |
| **Time to Resolution of DKA** | | | | | | | | | | | | |
| 6 | randomised trials | serious^a^ | not serious | not serious | not serious | none | 226 | 181 | - | MD **4.07 lower** (5.73 lower to 2.42 lower) | ⨁⨁⨁◯ Moderate^a^ |  |
| **Length of Hospital Stay** | | | | | | | | | | | | |
| 3 | randomised trials | serious^a^ | not serious | not serious | serious^b^ | none | 108 | 109 | - | MD **2.38 lower** (7.94 lower to 3.17 higher) | ⨁⨁◯◯ Low^a,b^ |  |
| **Rebound Hyperglycemia** | | | | | | | | | | | | |
| 5 | randomised trials | serious^a^ | not serious | not serious | not serious | none | 74/195 (37.9%) | 98/172 (57.0%) | **RR 0.70** (0.46 to 1.07) | **171 fewer per 1,000** (from 308 fewer to 40 more) | ⨁⨁⨁◯ Moderate^a^ |  |
| **Recurrent DKA** | | | | | | | | | | | | |
| 3 | randomised trials | serious^a^ | not serious | not serious | not serious | none | 3/154 (1.9%) | 7/125 (5.6%) | **RR 0.47** (0.14 to 1.64) | **30 fewer per 1,000** (from 48 fewer to 36 more) | ⨁⨁⨁◯ Moderate^a^ |  |

**CI:** confidence interval; **MD:** mean difference; **RR:** risk ratio

#### Explanations

a. Several studies showed concerns in domains 2 and 4, and a downgrade was done for this reason.

b. We downgraded one level because the confidence interval is wide, and the sample size is smaller than the optimal information size

**Supplementary Table 2.** Detailed GRADE assessment of included outcomes, including risk of bias, inconsistency, indirectness, imprecision, and publication bias.
